# Strong Axiality in a Dysprosium(III) Bis(borolide) Complex Leads to Magnetic Blocking at 65 K

**DOI:** 10.1021/jacs.2c08568

**Published:** 2023-01-11

**Authors:** Alexandre H. Vincent, Yasmin L. Whyatt, Nicholas F. Chilton, Jeffrey R. Long

**Affiliations:** † Department of Chemistry, University of California, Berkeley, Berkeley, California94720, United States; ‡ Department of Chemistry, The University of Manchester, Oxford Road, ManchesterM13 9PL, U.K.; § Department of Chemical & Biomolecular Engineering, University of California, Berkeley, Berkeley, California94720, United States; ∥ Materials Sciences Division, Lawrence Berkeley National Laboratory, Berkeley, California94720, United States

## Abstract

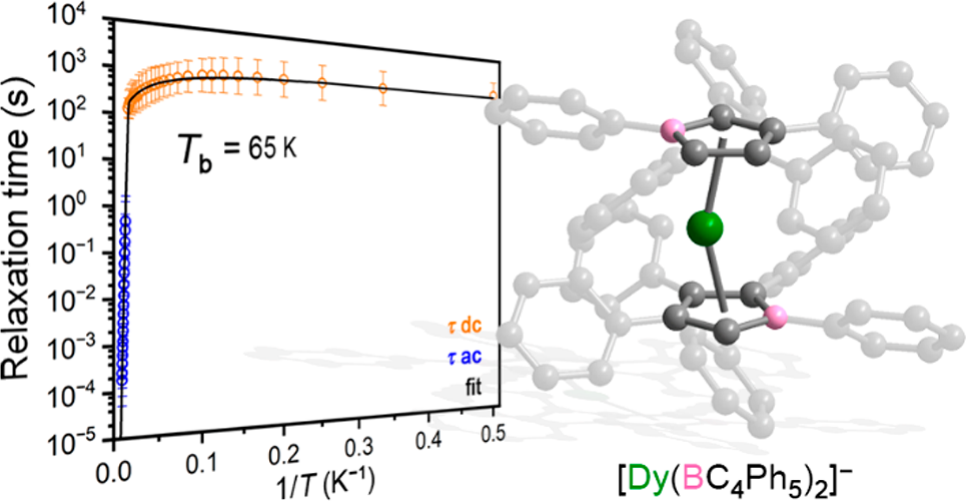

Substituted dysprosocenium complexes of the type [Dy(Cp^*R*^)_2_]^+^ exhibit slow magnetic relaxation at cryogenic temperatures and have emerged as top-performing single-molecule magnets. The remarkable properties of these compounds derive in part from the strong axial ligand field afforded by the cyclopentadiene anions, and the design of analogous compounds with even stronger ligand fields is one promising route toward identifying new single-molecule magnets that retain a magnetic memory at even higher temperatures. Here, we report the synthesis and characterization of a dysprosium bis(borolide) compound, [K(18-crown-6)][Dy(BC_4_Ph_5_)_2_] (**1**), featuring the dysprosocenate anion [Dy(BC_4_Ph_5_)_2_]^−^ with a pseudoaxial coordination environment afforded by two dianionic pentaphenyl borolide ligands. Variable-field magnetization data reveal open magnetic hysteresis up to 66 K, establishing **1** as a top-performing single-molecule magnet among its dysprosocenium analogues. Ac magnetic susceptibility data indicate that **1** relaxes via an Orbach mechanism above ∼80 K with *U*_eff_ = 1500(100) cm^–1^ and τ_0_ = 10^–12.0(9)^ s, whereas Raman relaxation and quantum tunneling of the magnetization dominate at lower temperatures. Compound **1** exhibits a 100 s blocking temperature of 65 K, among the highest reported for dysprosium-based single-molecule magnets. *Ab initio* spin dynamics calculations support the experimental *U*_eff_ and τ_0_ values and enable a quantitative comparison of the relaxation dynamics of **1** and two representative dysprosocenium cations, yielding additional insights into the impact of the crystal field splitting and vibronic coupling on the observed relaxation behavior. Importantly, compound **1** represents a step toward the development of alternatives to substituted dysprosocenium single-molecule magnets with increased axiality.

## Introduction

Slow magnetic relaxation of molecular origin can be observed in paramagnetic complexes possessing a bistable magnetic ground state. Known as single-molecule magnets, such compounds can exhibit magnetic memory effects at the single-molecule level.^1,2^ A characteristic of single-molecule magnets is an intrinsic energy barrier to magnetization reversal, similar to that occurring in superparamagnetic nanoparticles,^2^ which can be overcome by sequential spin-phonon excitations. Relaxation via this mechanism is known as the Orbach process, and the experimentally determined barrier is denoted *U*_eff_.^1^ Some of the most promising single-molecule magnets discovered to date are based on trivalent lanthanide ions, where the electronic states that define the *U*_eff_ barrier are the *M*_*J*_ levels of the ground Russell–Saunders spin–orbit term.^3^ The splitting and mixing of these *M*_*J*_ states, and hence the nature of the magnetic anisotropy, can been manipulated using coordination chemistry.^4^ As such, single-molecule magnets have attracted considerable attention for their potential use as a bit-patterned information storage medium as well as their potential utility in the nascent field of quantum information science.^5−7^

While lanthanide single-molecule magnets have been the subject of intense study for nearly two decades, only recently was it discovered that mononuclear compounds containing dysprosocenium cations can exhibit open magnetic hysteresis near liquid nitrogen temperatures,^8−10^ with 100 s blocking temperatures as high as *T*_b_ = 65 K in the case of [Cp^*i*Pr5^DyCp*][B(C_6_F_5_)_4_] (Cp^*i*Pr5^ = pentaisopropylcyclopentadienyl; Cp* = pentamethylcyclopentadienyl).^8^ These studies validated the notion that molecular magnets could retain magnetic memory at practical temperatures and have also contributed to the body of evidence showing that high-temperature hysteresis is not obtained solely through maximization of *U*_eff_.^11,12^ This general observation is illustrated through a comparison of the dysprosocenium compound [Dy(Cp^ttt^)_2_][B(C_6_F_5_)_4_] (Cp^ttt^ = 1,2,4-tri(*tert*-butyl)cyclopentadienyl)^13^ and the pentagonal bipyramidal complex salt [Dy(O^*t*^Bu)_2_(py)_5_][B(C_6_H_5_)_4_] (py = pyridine).^14^ Although the experimental relaxation barrier of the former is comparable to that of the latter compound (1223(28) versus 1251(14) cm^–1^, respectively),^15^ the dysprosocenium compound exhibits open magnetic hysteresis at much higher temperatures (up to 60 versus 14 K). In the case of [Dy(O^*t*^Bu)_2_(py)_5_][B(C_6_H_5_)_4_], additional magnetization reversal pathways, namely two-phonon (Raman) and quantum tunneling of magnetization processes,^1^ supersede Orbach relaxation at temperatures below 70 K and occur on faster time scales than in [Dy(Cp^ttt^)_2_][B(C_6_F_5_)_4_]. It has been shown that the remarkable performance of dysprosocenium-based compounds arises due to their strong axial ligand field, high-energy intramolecular vibrations, and low-energy intermolecular vibrations. The latter have the effect of suppressing the Raman relaxation mechanism,^16^ while it is speculated that the high-energy intramolecular vibrations also play a role in suppressing the quantum tunneling of magnetization.^17^

Recently, some of us have developed *ab initio* methods to understand the factors contributing to the very slow magnetic relaxation observed for dysprosocenium cations.^10,18−20^ These studies revealed that both a large crystal field splitting (with appropriate uniaxial magnetic anisotropy) and a low density of vibrational modes near electronic excitation energies are important characteristics. However, these computations also suggest that the upper limit of the relaxation barrier in biscyclopentadienyl dysprosium compounds may have already been achieved, and as such, enhancements in blocking temperatures within that family should focus on structural modifications to minimize the vibrational modes that are on resonance with electronic excitations.^19^ An alternative strategy is to target complexes analogous to the dysprosocenium compounds but possessing even stronger ligand fields in order to enhance the crystal field splitting, such as via heteroatom substitution in the cyclopentadienyl ring. To date, there is only one example of a dysprosium sandwich complex featuring a five-membered heterocycle, namely [Dy(P(C^*t*^BuCMe)_2_)_2_][B(C_6_F_5_)_4_],^19^ which features substituted phospholyl ligands and exhibits similar performance to some dysprosocenium single-molecule magnets^10^ with a *U*_eff_ = 1220(50) cm^–1^ and a 100 s blocking temperature of 23 K.^20^

In considering alternatives to the substituted dysprosocenium archetype, we sought to identify heterocycles that could provide structural rigidity and a stronger ligand field. Inspired by the linear divalent lanthanide metallocenes Ln(Cp^Ph5^)_2_ (Ln = Eu, Yb; Cp^Ph5^ = pentaphenylcyclopentadienyl),^21,22^ we hypothesized that a dysprosium sandwich complex featuring the dianionic pentaphenylborolide (BC_4_Ph_5_)^2–^ ligand^23^ might exhibit an even larger crystal field splitting—and hence a larger barrier to magnetic relaxation—than members of the dysprosocenium family, as a result of the larger negative charge on the ligands. In addition, the phenyl substituents might lead to higher-energy intramolecular C–C vibrational modes than characterized for dysprosocenium compounds with alkyl substituents. Here, we present the synthesis and characterization of [K(18-crown-6)][Dy(BC_4_Ph_5_)_2_] (**1**), featuring such an anionic Dy^III^ sandwich complex. Of note, while the present work was under review, a related study was published on the magnetic properties of the compound [K(2.2.2-cryptand)][Dy(BC_4_Ph_4_Pip)_2_] ((BC_4_Ph_4_Pip)^2–^ = 1-(piperidino)-2,3,4,5-tetraphenylborolide),^24^ which features a similar anionic Dy^III^ bis(borolide) sandwich complex. We find that our compound **1** exhibits a large relaxation barrier of *U*_eff_ = 1500(100) cm^–1^ and a 100 s blocking temperature of *T*_b_ = 65 K that are among the highest reported to date among dysprosium single-molecule magnets and comparable with the magnitudes of *U*_eff_ and *T*_b_ reported for [K(2.2.2-cryptand)][Dy(BC_4_Ph_4_Pip)_2_].^8,24,25^

## Results and Discussion

### Synthesis and Structural Characterization

**Figure 1:**
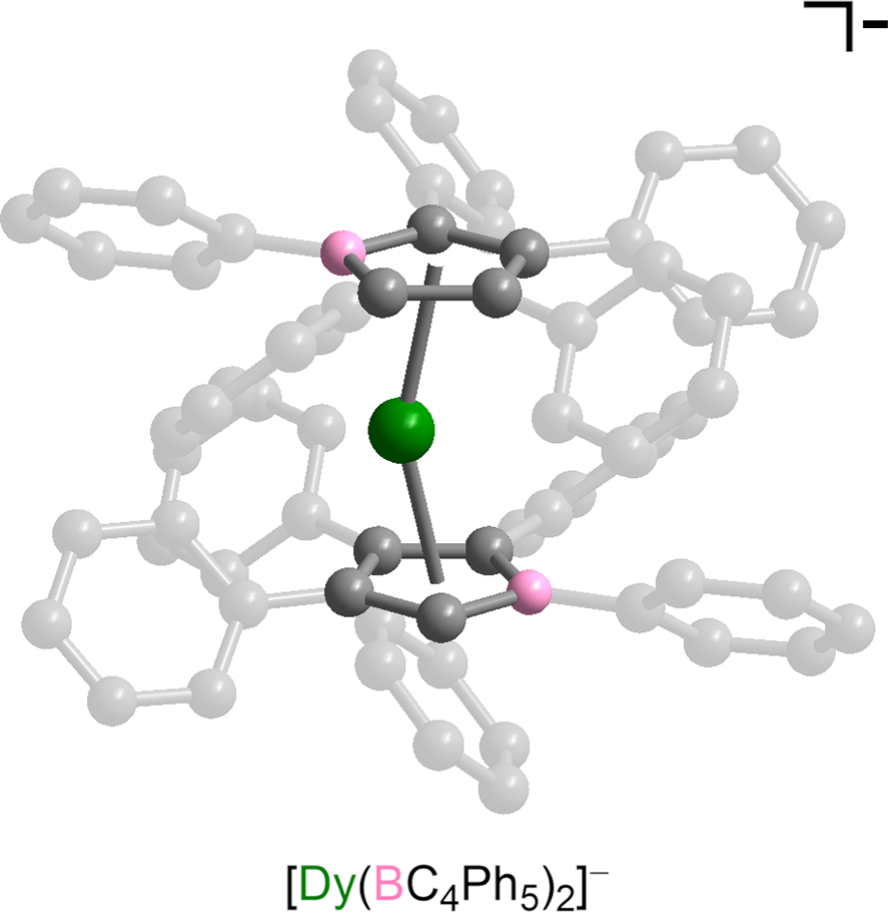
Structure of one of the unique [Dy(BC_4_Ph_5_)_2_]^−^ anions in the structure of **1**·2THF as determined via single-crystal X-ray diffraction. Green, gray, and pink spheres represent Dy, C, and B atoms, respectively. Hydrogen atoms, the lower occupancy dysprosium site, inversion-related dysprosium sites, and the [K(18-crown-6)(THF)_2_]^+^ cation are omitted for clarity. Selected interatomic distances and angles are provided in Table S2.

**Scheme 1:**
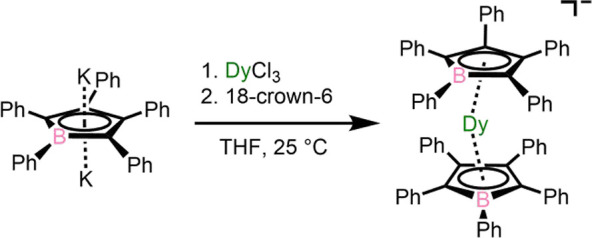
Synthesis of **1**·2THF from Potassium Pentaphenyl Borolide and DyCl_3_ The [K(18-crown-6)(THF)_2_]^+^ countercation in the product is not shown.

The compound [K(18-crown-6)][Dy(BC_4_Ph_5_)_2_] (**1**) was synthesized via salt metathesis of anhydrous DyCl_3_ with K_2_BC_4_Ph_5_ in tetrahydrofuran (THF) followed by the addition of a small stoichiometric excess of 18-crown-6 (Scheme 1; see the Supporting Information for details). Dark red parallelepiped crystals were grown by layering a concentrated THF solution of the crude compound with diethyl ether. Single-crystal X-ray diffraction analysis revealed these crystals to be the solvated compound [K(18-crown-6)(THF)_2_][Dy(BC_4_Ph_5_)_2_] (**1**·2THF; Figure 1), in which two THF molecules are axially bound to the [K(18-crown-6)]^+^ countercation.

The compound **1**·2THF crystallizes in the space group *P*–1 with two unique complex ions in the unit cell, each having identical connectivity but with slightly different bond lengths and angles (see Table S2). The Dy^III^ center in each unique anionic complex is disordered about a crystallographic inversion center located at the center of the molecule. For each unique anion, two partially occupied dysprosium sites were identified with variable occupancies totaling to 0.5. Applying the inversion operation yielded two more symmetry equivalent positions for each anion, affording a total Dy occupancy across all disordered positions of ∼1. The average Dy–centroid distance is 2.326 Å, and the average centroid–Dy–centroid bend angle is 156.5°, within the range of reported values (2.273(7)–2.358(6) Å and 146.4(5)°–162.50(12)°, respectively) for substituted dysprosocenium cations.^8,9,13^ In the structure of [K(2.2.2-cryptand)][Dy(BC_4_Ph_4_Pip)_2_], the dysprosium(III) was modeled as disordered over two sites with average Dy–centroid distances and centroid–Dy–centroid bend angles of 2.259 Å and 161.4(3)° and 2.269 Å and 158.6(3)°, respectively, which are slightly larger than those determined for **1**·2THF.^24^ We ascribe these differences to the greater electron-donating ability of piperidyl over phenyl and the greater overall steric bulk of (BC_4_Ph_4_Pip)^2–^ relative to (BC_4_Ph_5_)^2–^. Noncovalent edge-to-face interactions between phenyl groups of the individual (BC_4_Ph_5_)^2–^ ligands in **1**·2THF are also apparent from close edge-to-face distances. These interactions, in concert with the steric bulk of the ligands, stabilize the desired axial coordination geometry. We note that all magnetic characterization data described below were collected for a sample of **1**·2THF dried under reduced pressure for a minimum of 30 min to remove bound solvent. This was done to avoid any uncertainty in the bulk composition (molecular mass) of the sample being measured and therefore the quantitative magnetic results (see Section 1.6 of the Supporting Information). Inductively coupled plasma optical emission spectrometry analysis of a sample prepared in this way revealed Dy, K, and B content consistent with the fully desolvated formula [K(18-crown-6)][Dy(BC_4_Ph_5_)_2_] (**1**). The local coordination environment around the Dy^III^ centers is not expected to change substantially with loss of the two outer-sphere THF molecules, and therefore we expect there to be limited impact on the magnetic properties (see the discussion of the magnetic properties below for additional details).

### Electronic Structure Calculations

State-average complete active space self-consistent field spin-orbit (CASSCF-SO) calculations were performed in OpenMolcas^26^ using the single-crystal structure of one of the [Dy(BC_4_Ph_5_)_2_]^−^ anions in **1**·2THF; the active space comprised nine electrons in seven 4f orbitals of Dy^III^. These calculations predict that the crystal field splitting of the ^6^H_15/2_ ground state is approximately 1800 cm^–1^, which is comparable with that predicted for the two disordered components in [K(2.2.2-cryptand)][Dy(BC_4_Ph_4_Pip)_2_]^24^ (1713 and 1638 cm^–1^) and slightly smaller than the total crystal field splitting of 2104 cm^–1^ calculated for [Cp^*i*Pr5^DyCp*][B(C_6_F_5_)_4_].^8^ Projecting the spin–orbit states onto a crystal field Hamiltonian with SINGLE_ANISO,^27^ the calculations predict a pure *M*_*J*_ = ±15/2 ground state (eas*y*-axis magnetic anisotropy; see Tables S8 and S9), and the first four excited state terms (well-approximated by *M*_*J*_ = ±13/2, ±11/2, and ±9/2, respectively) are also highly axial; however, the effective *g* values of the remaining Kramers doublets are significantly nonaxial. On the basis of the calculated energies of the sixth Kramers doublet from two different levels of theory, *U*_eff_ is predicted to be between 1420 and 1480 cm^–1^.

### Magnetic Properties

**Figure 2:**
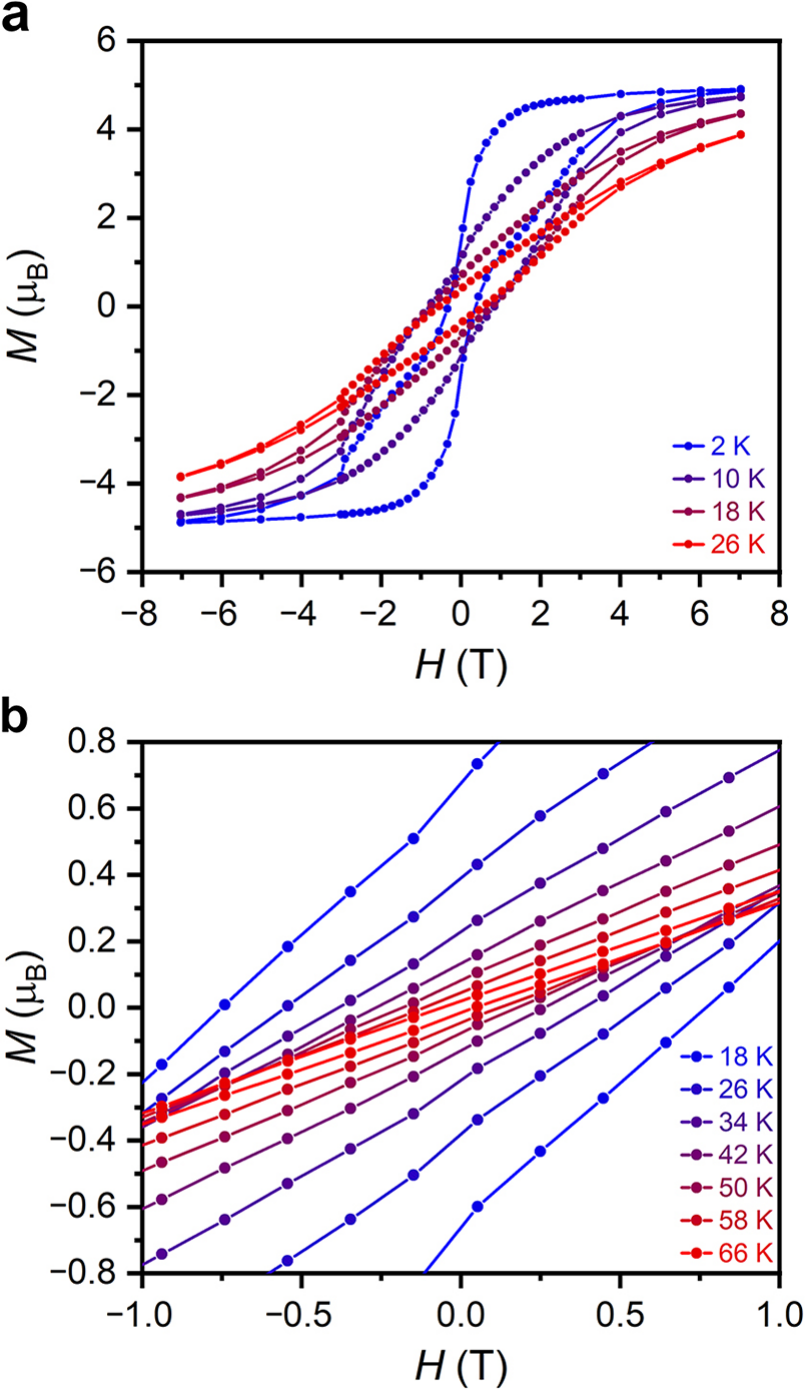
(a) Variable-field magnetization curves for **1** at select temperatures. A sweep rate of 84 Oe/s was used for data collected below 3 T, whereas a sweep rate of 165 Oe/s was used for data collected above 3 T. (b) Expanded view of the variable-field magnetization curves for **1** between ±1 T at the indicated temperatures.

**Figure 3:**
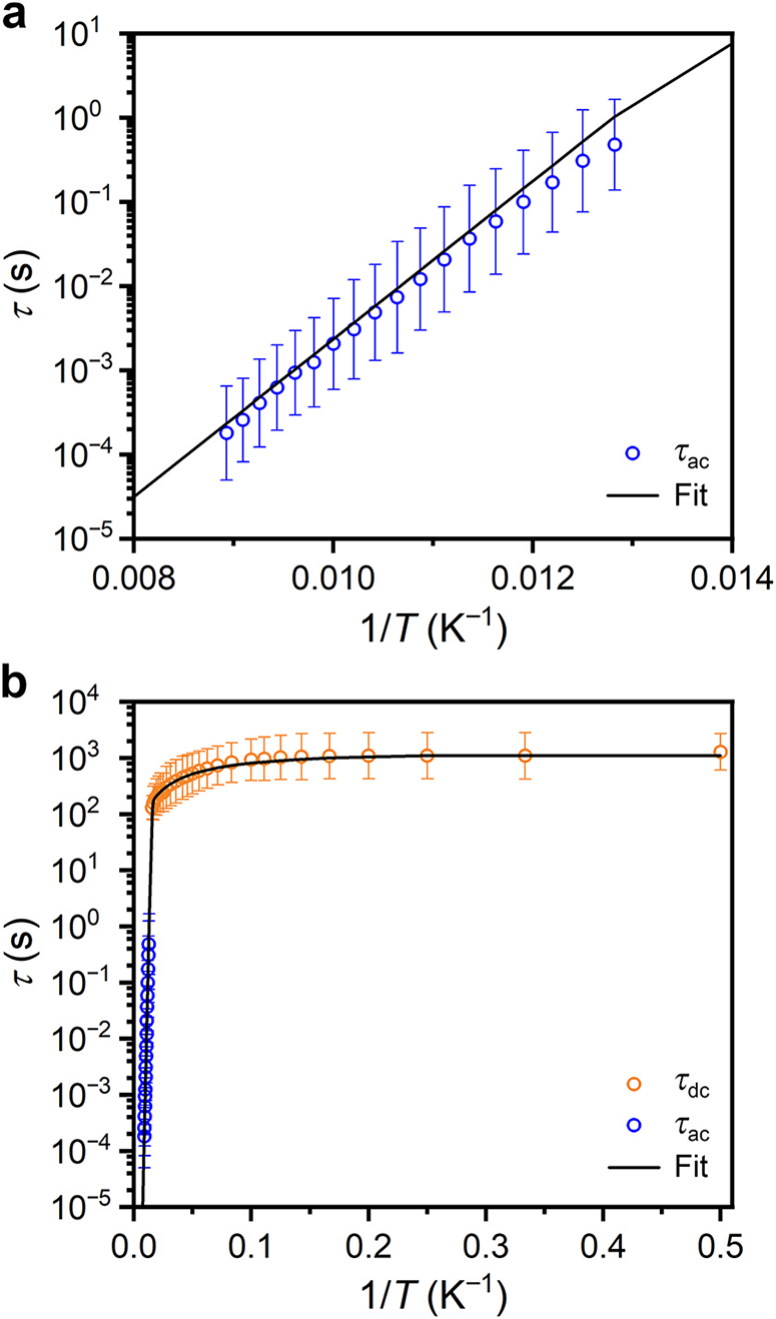
(a) Plot of the magnetic relaxation times (τ, log scale) versus 1/*T* determined for **1** from ac susceptibility data (blue symbols) and corresponding fit using eq 1 as described in the text. Estimated standard deviations at the 1σ level from the generalized Debye model are indicated with error bars.^15^ (b) Plot of the magnetic relaxation times (τ, log scale) versus 1/*T* determined for **1** from dc relaxation data (orange symbols) and ac susceptibility data (blue symbols) and corresponding fit using eq 1 as described in the text. Estimated standard deviations at the 1σ level for the ac data are from the generalized Debye model and indicated with error bars.^15^ Estimated standard deviations at the 1σ level for the dc data were calculated according to the empirical formula given in Table S3.^20^

Variable-field magnetization data were collected for **1** between ±7 T as an initial probe of slow magnetic relaxation. Open-loop magnetic hysteresis was apparent at temperatures as high as 66 K (Figure 2), comparable to the highest hysteresis temperature of 70 K reported for [K(2.2.2-cryptand)][Dy(BC_4_Ph_4_Pip)_2_] (determined using a sweep rate of 9 Oe/s).^24^ At 2 K, the slightly waist-restricted hysteresis may arise due to quantum tunneling of the magnetization. The magnetic remanence and coercive field at this temperature are 1.77 μ_B_ and 0.34 T, respectively. Frequency-dependent ac magnetic susceptibility data were collected for **1** to investigate the mechanism(s) of magnetic relaxation at higher temperatures (see the Supporting Information for details). Under zero dc field, peaks were apparent in the out-of-phase magnetic susceptibility at temperatures ranging from 78 to 112 K. Plots of the molar in-phase (χ_M_*′*) and out-of-phase (χ_M_″) components of the ac susceptibility versus frequency (Figures S18 and S19) were fit to the generalized Debye model^15^ to extract temperature-dependent relaxation times (τ). As shown in Figure 3a, the resulting plot of τ (natural log scale) versus *T* (inverse scale) is approximately linear, indicating relaxation via an Orbach mechanism.^28^

Temperature-dependent dc magnetic relaxation measurements were also performed to determine relaxation times at lower temperatures from 2 to 64 K. These data could be adequately fit using a stretched exponential function (see Figures S12–S14, Table S3, and the Supporting Information for details), which is frequently employed to extract relaxation times from magnetization decay data.^2,28^ The τ values derived from the dc relaxation measurements (log scale) are plotted versus 1/*T* in Figure 3b along with the corresponding data from the ac relaxation measurements. Below ∼10 K the data are nearly temperature-independent, consistent with relaxation via quantum tunneling of the magnetization. Between 10 and 64 K, the relaxation times for each process exhibit a weak temperature dependence consistent with a two-phonon Raman process.
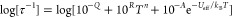
1The combined relaxation times obtained from the ac susceptibility and dc magnetic relaxation data were fit with CCFIT2^15^ using eq 1 (where *A* and *Q* are the negative logarithms of the attempt time (τ_0_) and tunneling time (τ_tun_), respectively, and *C* and *n* are the Raman prefactor exponent and temperature exponent, respectively) to give *U*_eff_ = 1500(100) cm^–1^, τ_0_ = 10^*A*^ = 10^–12.0(9)^ s, *C* = 10^*R*^ = 10^–5(1)^ s^–1^ K^–*n*^, *n* = 1.3(8), and τ_tun_ = 10^*Q*^ = 10^3.1(3)^ s (Figure 3, black curve). Importantly, the fitted value of *U*_eff_ is in very good agreement with that predicted from CASSCF-SO calculations using the optimized structure of **1**·2THF, supporting our assertion that desolvation should not significantly impact the magnetic properties. We also attempted to prepare a magnetic sample of the pristine solvated compound, **1**·2THF, immediately following recrystallization and after only brief (a few seconds) exposure to dynamic vacuum to remove crystallization solvent. However, dc magnetic susceptibility data collected for this sample (see Figures S10 and S11) suggest that the sample readily desolvates (see Section 1.6 of the Supporting Information for details). Even still, a suite of magnetic data collected for this second sample (see Tables S4, S6, and S7; Figures S10, S11, S15–S17, and S21–S24) support the reproducibility of the data collected for **1**. Indeed, the fit parameters (*U*_eff_ = 1600(100) cm^–1^, τ_0_ = 10^*A*^ = 10^–12.6(8)^ s, *C* = 10^*R*^ = 10^–5(2)^ s^–1^ K^–*n*^, *n* = 1(1), and τ_tun_ = 10^*Q*^ = 10^3.1(3)^ s) are within error of those determined for **1**.

The estimated *U*_eff_ = 1500(100) cm^–1^ for **1** is among the highest values reported to date for dysprosium-containing single-molecule magnets^8−10,19^ and comparable to the magnitudes of the two *U*_eff_ values reported for [K(2.2.2-cryptand)][Dy(BC_4_Ph_4_Pip)_2_] (1600(100) and 1300(300) cm^–1^), which were ascribed to the two disordered components in the structure.^24^ Interestingly, quantum tunneling in **1** is at least an order of magnitude faster than in [Cp^*i*Pr5^DyCp*][B(C_6_F_5_)_4_] (τ_tun_ ∼ 10^4^ s), as suggested by the narrowed hysteresis loops observed for compound **1** (Figure 2a). The Raman exponent is quite small but similar in magnitude to those determined for dysprosocenium cations and [K(2.2.2-cryptand)][Dy(BC_4_Ph_4_Pip)_2_].^10,16,24^ Such low values arise in the so-called “high-temperature” limit of traditional approximations to the Raman mechanism, where the Debye frequency of the lattice acoustic phonons is larger than *k*_B_*T*/ℏ.^16,29^

In addition to *U*_eff_, another metric often used to compare single-molecule magnets is the so-called magnetic blocking temperature (*T*_b_), which has been defined as the temperature at which the magnetic relaxation time is equal to 100 s.^2^ From the fit of the ac and dc relaxation data for **1**, we can estimate *T*_b_ = 65 K, which is among the highest values reported to date for a dysprosium single-molecule magnet. The same blocking temperature was reported for [Cp^*i*Pr5^DyCp*][B(C_6_F_5_)_4_] (*T*_b_ = 65 K),^8^ while a 100 s blocking temperature of 66 K was determined for [K(2.2.2-cryptand)][Dy(BC_4_Ph_4_Pip)_2_.^24^ To our knowledge, the only compound to exhibit a higher 100 s blocking temperature is the mixed-valent dinuclear complex (Cp^iPr5^)_2_Dy_2_I_3_, with *T*_b_ = 72 K.^25^

### Computational Analysis of Relaxation Dynamics

To gain insight into the molecular origin of the high-temperature relaxation in **1**, we performed spin-dynamics calculations using a method previously described by some of us.^19^ Gas-phase geometry optimizations and vibrational normal mode calculations were performed on one of the [Dy(BC_4_Ph_5_)_2_]^−^ anions in **1**·2THF using Gaussian 09d,^30^ where the PBE (leading to structure **1-PBE**) and PBE0 (leading to structure **1-PBE0**) density functionals and Grimme’s empirical dispersion correction were employed.^31−33^ The root-mean-square deviations between the optimized [Dy(BC_4_Ph_5_)_2_]^−^ structures and the experimental structure were found to be only 0.245 and 0.236 Å for **1-PBE** and **1-PBE0**, respectively. We then used OpenMolcas^26^ to perform state-average CASSCF-SO calculations on the optimized equilibrium structures of the anion (active space: nine electrons in seven 4f orbitals) and projected the spin–orbit states onto a crystal field Hamiltonian.^27^ The spin-phonon coupling coefficients of each vibrational mode were determined by distorting the equilibrium geometry of **1-PBE** and **1-PBE0** along their normal mode coordinates in positive and negative directions and performing CASSCF-SO calculations to obtain the derivatives of the crystal field parameters via finite differences.

**Figure 4:**
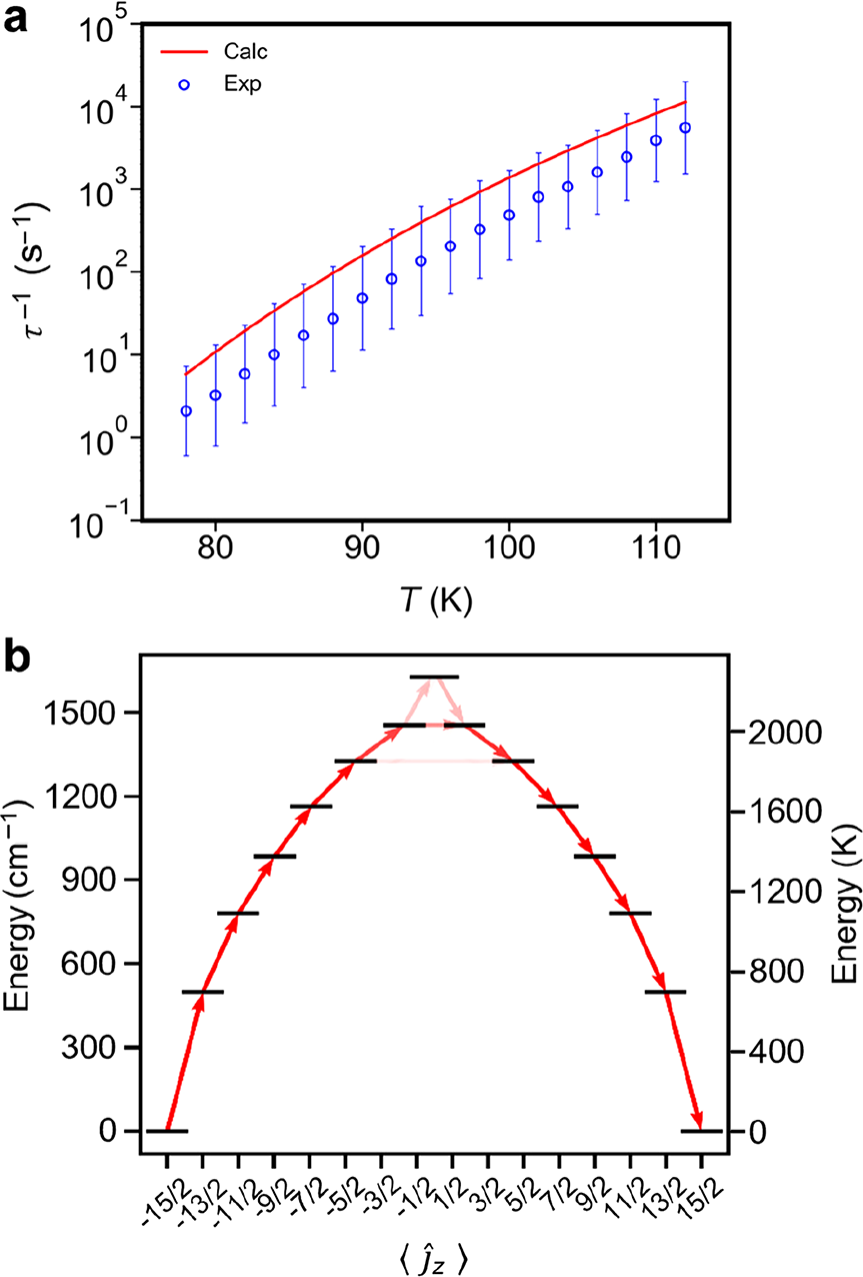
(a) Temperature dependence of calculated (red line, FWHM = 10 cm^–1^, **1-PBE**) and experimental (blue symbols) relaxation rates. Error bars are 1σ estimated standard deviations determined from the generalized Debye model.^15^ (b) Electronic states of the eight Kramers doublets of the ground ^6^H_15/2_ multiplet of **1-PBE**. Red arrows represent relaxation pathways, where the opacity of each arrow is proportional to the normalized spin-phonon transition probability.

With the spin-phonon coupling coefficients in hand, we calculated magnetic relaxation times using Fermi’s Golden Rule.^19^ Here, the only free parameter is the full width at half-maximum (FWHM) vibrational line width, and we considered values of 5, 10, and 20 cm^–1^. The relaxation rate was generally found to increase with increasing line width (Figure S27) due to more modes coming into resonance with the relevant electronic transitions. The calculated relaxation rates are in good agreement with experimental data (Figures 4a and S27). In particular, at a fixed line width of 10 cm^–1^, the results for **1-PBE0** are in excellent agreement with experiment (Figure S28). The results for **1-PBE** would only require a slight decrease in the FWHM line width in order to bring them to the same level of agreement (Figure 4a), but we do not perform such an optimization here. Instead, to be consistent with our previous work, we will here focus on results using the PBE density functional and a FWHM line width of 10 cm^–1^.^19^ On the basis of the results of our *ab initio* spin-phonon coupling calculations, relaxation via the Orbach mechanism likely proceeds via the fifth or sixth excited Kramers doublets, as illustrated in the diagram in Figure 4b.

In order to facilitate a more direct comparison of factors dictating Orbach relaxation in **1** and dysprosocenium complexes, we selected [Cp^*i*Pr5^DyCp*][B(C_6_F_5_)_4_]^8^ and [Dy(Cp^*i*Pr4^)_2_][B(C_6_F_5_)_4_],^9^ which exhibit the slowest and fastest relaxation rates in the Orbach region for this family of compounds. A comparison of the experimental temperature-dependent relaxation times revealed that compound **1** exhibits faster relaxation than the former but slower relaxation than the latter compound (Figure S29a), and this ordering is also borne out by our *ab initio* calculations (Figure S29b). To gain insight into this result, we performed a mode-weighted analysis comparing **1** with these two dysprosocenium compounds (see Section 2.4 of the Supporting Information). Briefly, in this approach, we decompose the relaxation rate matrix of a compound into its components (spin-phonon coupling ⟨*H̿*_SP_⟩, phonon occupation ⟨*Q̿*⟩, phonon density of states ⟨ρ̿⟩, and average number of phonon modes ⟨*n̿*⟩), substitute one of these components for the equivalent component from another compound, solve the master equation by diagonalizing the rate matrix, and assesses how this changes the relaxation rate. However, as the rates are eigenvalues of the rate matrix, substitution of components does not always result in exact reciprocity. For example, substituting the spin-phonon coupling ⟨*H̿*_SP_⟩ from molecule X into the rate matrix for molecule Y may increase the relaxation rate by an order of magnitude, but one may not observe the reciprocal numerical decrease in the relaxation rate if the ⟨*H̿*_SP_⟩ from molecule Y is used for molecule X. Hence, the safest analysis is made by looking at the largest changes, and for consistency in the direction of changes under reciprocal swaps.

The largest change for any swap between **1** and [Cp^*i*Pr5^DyCp*][B(C_6_F_5_)_4_] occurs when ⟨*H̿*_SP_⟩ from **1** is used for [Cp^*i*Pr5^DyCp*][B(C_6_F_5_)_4_], rendering relaxation in [Cp^*i*Pr5^DyCp*][B(C_6_F_5_)_4_] approximately 5 times faster; the reciprocal swap (⟨*H̿*_SP_⟩ from [Cp^*i*Pr5^DyCp*][B(C_6_F_5_)_4_] into **1**) results in a decrease in the relaxation rate of **1** by a factor of ∼2 (Table S17). These changes are consistent in their direction and with the overall difference in relaxation rates between the two compounds (Figure S29); swapping other components has a smaller effect and also shows nonreciprocity. Hence, this analysis suggests that compound **1** exhibits faster relaxation than [Cp^*i*Pr5^DyCp*][B(C_6_F_5_)_4_] due to overall stronger spin-phonon coupling. Comparing **1** with [Dy(Cp^*i*Pr4^)_2_][B(C_6_F_5_)_4_], the largest change is observed when the phonon occupation ⟨*Q̿*⟩ from **1** is substituted into [Dy(Cp^*i*Pr4^)_2_][B(C_6_F_5_)_4_], which renders relaxation in [Dy(Cp^*i*Pr4^)_2_][B(C_6_F_5_)_4_] 14 times slower; the reciprocal swap (when ⟨*Q̿*⟩ is swapped from [Dy(Cp^*i*Pr4^)_2_][B(C_6_F_5_)_4_] into **1**) results in nearly an order of magnitude increase in the relaxation of **1**; all other swaps have a far less significant effect (Table S18). Hence, this analysis suggests that **1** is a higher-performance single-molecule magnet than [Dy(Cp^*i*Pr4^)_2_][B(C_6_F_5_)_4_] because of its larger crystal field splitting.^19^

While our original hypothesis was that the formally dianionic borolide ligands could engender a larger crystal field splitting in the ground state of dysprosium(III)—and therefore give rise to a larger *U*_eff_—than monoanionic cyclopentadienide ligands, the performance of **1** is similar to its dysprosocenium relatives. Given the similar structural metrics between the dysprosocenate anion in **1** and known dysprosocenium cations, this suggests that the effective charges on the ligands are also similar. To explore this possibility, we performed DFT calculations on the isolated ligands (BC_4_Ph_5_)^2–^, (Cp^ttt^)^−^, (Cp^iPr5^)^−^, (Cp*)^−^, and [P(C^t^BuCMe)_2_]^−^ in the gas phase and used Löwdin population analysis to infer effective atomic charges (see the Supporting Information for details). These calculations give aggregate charges on the five-membered rings of −0.71, −0.90, −0.60, −0.68 and −0.78, respectively (Tables S19 and S20). It is clear from these data that the BC_4_ ring in (BC_4_Ph_5_)^2–^ does not hold double the anionic charge compared to its monoanionic counterparts and that the phenyl rings withdraw significant charge (approximately −0.2 to −0.3, Table S19). Hence, along with the structural similarities described above, these data serve to explain the similar performance of **1** compared to dysprosocenium-based single-molecule magnets. Our results also suggest that the design of dysprosium bis(borolide) complexes featuring ligands with electron-donating groups is a worthwhile pursuit.

## Conclusions

We have detailed the synthesis and characterization of a new dysprosium(III) compound featuring dianionic borolide ligands, [K(18-crown-6)][Dy(BC_4_Ph_5_)_2_] (**1**), which is a single-molecule magnet with performance comparable to the state-of-the art dysprosocenium compounds and the recently discovered dysprosium(III) bis(borolide) complex [Dy(BC_4_Ph_4_Pip)_2_]^−^.^24^ In particular, compound **1** displays magnetic hysteresis with a 100 s magnetic blocking temperature of 65 K, and analysis of ac magnetic susceptibility data showed it to exhibit a single Orbach relaxation process with *U*_eff_ = 1500(100) cm^–1^ and τ_0_ = 10^–*A*^ = 10^–12.0(9)^ s. *Ab initio* calculations of the spin dynamics of **1** predicted relaxation rates in the Orbach regime in excellent agreement with the experimentally determined rates. In addition, our mode-weighted analysis suggests that although the crystal field splitting of **1** is slightly smaller than that of the top-performing dysprosocenium single-molecule magnet [Cp^*i*Pr5^DyCp*][B(C_6_F_5_)_4_],^8^ stronger spin-phonon coupling in **1** is the more important factor influencing its faster relaxation dynamics. As such, an exciting area of future investigation will be the synthesis and study of other substituted dysprosocenate complexes with the goal of identifying substituents that will minimize spin-phonon coupling in this system. In addition, our DFT calculations suggest that enhancement of the crystal field splitting in dysprosium bis(borolide) complexes may also be achieved through judicious choice of electron-donating functional groups. Notably, there are a large number of reported substituted boroles,^34,35^ which could conceivably undergo reduction and subsequent metalation with Dy^III^. As a result of the more diverse substitution chemistry of the borole heterocycle relative to cyclopentadiene, a potentially large and diverse family of new dysprosocenate compounds featuring strongly axial borolide ligands is accessible.

## Supporting Information Available

Supplementary structural, spectroscopic, magnetic, and computational data (PDF)
