# Supplementary material for: Strong Axiality in a Dysprosium(III) Bis(borolide) Complex Leads to Magnetic Blocking at 65 K
Source: J Am Chem Soc. 2023 Jan 11;145(3):1572–9. doi: 10.1021/jacs.2c08568 (PMC9923674; doi:10.1021/jacs.2c08568)
Supplement: Supplementary file 1 — ja2c08568_si_001.pdf [file ja2c08568_si_001.pdf]

Supporting Information for:

Strong Axiality in a Dysprosium(III) Bis(borolide) Complex Leads to Magnetic Blocking at 65 K

Alexandre Vincent[a], Yasmin L. Whyatt[b], Nicholas F. Chilton[b*], and Jeffrey R. Long[a,c,d*]

[a] Department of Chemistry, University of California, Berkeley, California 94720, United States
[b] School of Chemistry, The University of Manchester, Oxford Road, Manchester M13 9PL
[c] Department of Chemical and Biomolecular Engineering, University of California, Berkeley, California 94720, United States
[d] Materials Sciences Division, Lawrence Berkeley National Laboratory, Berkeley, California 94720, United States

Table of Contents

1. Experimental Methods 3

1.1. Material Preparation 3

1.2. Synthesis of [K(18-crown-6)][Dy(BC_4Ph_5)$_2$] (1) 3

1.3. Single Crystal X-Ray Diffraction Analysis 3

1.4. Inductively Coupled Plasma–Optical Emission Spectrometry 4

1.5. Fourier Transform Infrared Spectroscopy 4

1.6. Magnetometry 4

1.7. Nuclear Magnetic Resonance Spectroscopy

1.8. Powder X-Ray Diffraction

2. Computational Methods 5

2.1. Density Functional Theory (DFT) Calculations 5

2.2. CASSCF-SO Calculations 5

2.3. Spin Dynamics Calculations 6

2.4. Mode-Weighted Analysis 6

3. Figures and Tables 7

4. References 49

1. Experimental Methods

1.1. Material Preparation

All manipulations were carried out under an atmosphere of argon with rigorous exclusion of air and water using either standard Schlenk line techniques or a glovebox. Commercial reagents were used directly without further treatment unless otherwise noted. Anhydrous $DyCl_3$ was purchased from Strem Chemicals. The ligand $K_2BC_4Ph_5$ was synthesized from $PhBC_4Ph_4$ and potassium metal strips following literature procedure.[1] The ligand precursor $PhBC_4Ph_4$ was synthesized according to the literature method[2] from $(LiC_2Ph_2)_2$ and dichlorophenylborane (synthesized according to the literature method[3] from 1M BCl_3 in heptane (Millipore-Sigma) and tetrapheyltin (Alfa-Aesar)). The reagent $(LiC_2Ph_2)_2$ was synthesized from diphenylacetylene (Alfa-Aesar) and lithium metal strips according to the literature method.[4] Lithium and potassium metal pieces were flattened and cut into strips (~ 20 × 5 × 2 mm) prior to their addition to reaction mixtures. Tetrahydofuran (THF), benzene, toluene, and diethyl ether were dried and degassed using a JC Meyer solvent purification system and stored under Argon over 4 Å molecular sieves prior to use. A diethyl ether solution of 18-crown-6 was dried over 4 Å sieves for three days prior to use. The diethyl ether was then removed under reduced pressure to furnish solid, anhydrous 18-crown-6.

1.2. Synthesis of [K(18-crown-6)][Dy(BC_4Ph_5)$_2$] (1). In the glovebox, colorless anhydrous $DyCl_3$ (30.5 mg, 0.113 mmol) and THF (10 mL) were added to a 20 mL scintillation vial equipped with a stir bar, and the slurry was stirred for several minutes. The resulting suspension was then added quickly to a 20 mL scintillation vial containing red $K_2BC_4Ph_5$ powder (118 mg, 0.226 mmol, 2.00 equiv). The mixture was allowed to stir for 18 h at ambient temperature. Following the reaction, the mixture was centrifuged to separate the insoluble colorless solid, presumed to be mostly KCl with possible traces of unreacted $DyCl_3$. The solution was decanted into a separate scintillation vial. The solvent was removed from this solution under vacuum to yield a red solid residue and the residue was washed once with diethyl ether. A solution of 18-crown-6 (37 mg, 0.140 mmol) in THF (5 mL) was then added to the solid. The mixture was agitated until complete dissolution was achieved. The red color of the solution became more intense than it had been following centrifugation. The THF solvent was then removed under vacuum, and the resulting solid dark red residue was washed with diethyl ether (1 mL), and the resulting powder redissolved in THF (1 mL). This solution was layered beneath two volume equivalents of diethyl ether and left to recrystallize at room temperature. After standing for one day, dark red crystals of **1**·2THF had formed, which were suitable for single-crystal diffraction. Dry yield of **1**: 32.0 mg (21%). All attempts to obtain CHN analysis on both **1** and **1**·2THF failed. ICP-OES was used to establish compositional purity of **1**. Dy, K, B ICP-OES. Expected for $C_{80}H_{74}B_2DyKO_6$: 12.0% Dy, 2.89% K, 1.60% B; Found: 11.52% Dy, 3.05% K, 1.34% B. FT-IR neat: 405(w), 413(m), 420(m), 426(m), 440(w), 458(m), 464(m), 474(m), 479(m), 491(m), 499(m), 507(m), 527(m), 532(m), 542(m), 615(w), 646(m), 666(m), 699(s), 731(m), 768(m), 789(m), 834(m), 854(w), 867(w), 872(w), 884(w), 902(m), 926(w), 960(s), 979(w), 997(m), 1026(m), 1071(m), 1101(s), 1247(m), 1284(m), 1350(m), 1442(w), 1453(w), 1458(w), 1474(w), 1485(m), 1570(m), 1588(m), 2872(m), 2895(m).

1.3. Single Crystal X-Ray Diffraction Analysis

Crystals coated in Paratone-N oil were mounted onto MiTeGen 10 μm loops and moved under an Oxford Cryosystems nitrogen cryostat. Crystals were found to be very sensitive to oxygen, even under Paratone-N oil, decomposing into colorless material after only a few minutes of sitting in oil under the microscope. The diffraction patterns were produced using synchrotron radiation at the Advanced Light Source; Beamline 12.2.1. Unit cell determination and spot integration was performed using Bruker AXS and SAINT.[5] Absorption corrections were applied to spot intensities using the SADABS package.[6] Structural solutions were obtained using the Intrinsic Phasing method as implemented in ShelXT.[7] Least Squares refinement as implemented in ShelXL was used to refine structural models against F^2.[8] Olex2 was used as a graphical front-end for structural

solution and refinement.[9] Boron positions were initially assigned by assigning all five ring atoms as carbon, allowing chemical occupancies of the five-membered ring atoms to vary, and then selecting the position with the lowest chemical occupancy value to assign as boron. During preliminary CIF validation, the Hirshfeld rigid-bond test[10] detected incorrectly modeled boron atoms, producing a B-level alert. This alert was no longer present when the B atoms were assigned correctly.

Two other level B alerts arose during final cif validation as follows.

PLAT230_ALERT_2_B Hirshfeld Test Diff for C025 --C029 . 7.4 s.u.
C025 and C029 correspond to meta and para carbon atoms on one of the phenyl rings of $BC_4Ph_5^{2-}$. These two atoms are likely disordered across two positions. Attempts to model this disorder using SPLIT+SAME commands resulted in an unstable refinement with wildly variable thermal displacement parameters and low chemical occupancy values for one of the disordered phenyl positions. This instability was unresponsive to restraints, and so the second disordered position was ultimately not modeled, given its apparent low chemical occupancy and the instability of its thermal displacement parameters.

PLAT230_ALERT_2_B Hirshfeld Test Diff for C02A --C02J . 11.6 s.u.
C02A and C02J correspond to meta and para carbon atoms on another one of the phenyl rings of $BC_4Ph_5^{2-}$. These two atoms are probably disordered across two positions. Attempts to model this disorder also resulted in an unstable refinement with wildly variable thermal displacement parameters and small chemical occupancy values for one of the disordered phenyl positions. Again, this instability was unresponsive to restraints. The second disordered position was not modeled, given its low chemical occupancy and the instability of its thermal displacement parameters.

1.4. Inductively Coupled Plasma–Optical Emission Spectrometry

Inductively coupled plasma–optical emission spectrometry was performed on a Perkin Elmer ICP Optima 7000. Commercial standard solutions (1000 ppm in 5% nitric acid) were purchased from Inorganic Ventures. Calibration curves were constructed using five standard solutions, each containing Dy, B and K. The Dy, B, and K concentrations for each of the five standard solutions are as follows: 0.1 ppm, 1 ppm, 5 ppm, 10 ppm and 15 ppm. Each standard also contained 0.1 ppm Y as an internal standard. A blank solution only containing 0.1 ppm Y was also prepared to set the zero-point of the calibration curve. Samples were fully digested overnight with 2 mL of piranha solution prepared from analytical grade 30% H_2O_2 (Millipore-Sigma) and reagent grade sulfuric acid (Fisher). It was found that digesting in acid without an oxidant failed to fully dissolve the boron containing organic material, leading to consistently low boron values. After adding the internal standard to the acid digest, the solution was diluted with 5% nitric acid to a nominal concentration of ~10 ppm prior to analysis. Calibration curves for all analytes had $R^2 > 0.999$.

1.5. Fourier Transform Infrared Spectroscopy

The Fourier-transform infrared (FTIR) spectrum of **1** was collected inside of an N_2 glovebox using neat crystalline powder and a Shimadzu IRSpirit FTIR spectrometer operating in ATR mode. Peaks were identified using the multiple peak fitting routine as implemented in Origin 2022.

1.6. Magnetometry

All magnetic measurements were carried out on a Quantum Design MPMS-XL SQUID magnetometer. Under the conditions used for magnetic sample preparation (described below), at least partial desolvation of the potassium cation is likely. Thus, in order to avoid ambiguity regarding the bulk composition of the sample and the quantitative magnetic results, a sample of **1**·2THF was fully desolvated before sample preparation. A crystalline sample of **1**·2THF was dried under vacuum for a minimum of 30 min, mechanically ground, and 21.4 mg loaded into a quartz tube (inner diameter 5 mm outer diameter 7 mm), covered with a solid layer of eicosane

(56.8 mg), and flame sealed under vacuum. The eicosane was subsequently melted at 40 °C in order to restrain the sample (prevent crystallite torquing) and to improve thermal conductivity between the sample and the environment. A second magnetic sample was prepared using a freshly prepared (and recrystallized) sample of **1**·2THF held under dynamic vacuum for only a few seconds, with the goal of removing surface crystallization solvent while retaining coordinated THF. The crystals were then crushed to form a microcrystalline powder (sample mass was 12.6 mg) and the sample prepared as described above (eicosane mass used was 47.0 mg). Work up of dc susceptibility data collected for this sample assuming the molecular weight for **1**·2THF (see Figures S10 and S11) revealed that the magnitude of the $\chi_M T$ product at 300 K is 17.7 emu·K/mol, larger than that determined for **1** (15.0 emu·K/mol) and predicted for a free Dy^{3+} (14.17 emu·K/mol), which suggests overestimation of the sample mass and likely desolvation of the sample. Even still, a full suite of ac and dc relaxation data collected for this second sample are consistent with those obtained for **1** and serve to validate the data for the desolvated compound. Diamagnetic corrections were calculated using Pascal's constants,[11] and were applied to all reported magnetic susceptibility values unless otherwise noted.

To collect isothermal magnetization decay data, the samples were initially magnetized under an applied magnetic field of 7 T for 5 minutes and then the field set to zero, and magnetization measured for ca. 1600 s. However, we found there was a trapped field in the superconducting magnet, and magnetization decay curves at 64, 68 and 72 K fully equilibrated during the measurement time to a negative magnetization value. These negative equilibrium magnetization values were used to fit a Curie law $M(T) = C/T$ with $C = -0.00232$ emu K; this corresponds to a field of approximately −12 Oe. Decay data for 64 K and below were fit using eq S1,[12] where M_{eq} was fixed to $M_{\mathrm{eq}} = -0.00232/T$, M_0 is the first measured data point (set as time zero), t is the time after the first measured point M_0, τ is the characteristic relaxation time, and β is a stretching parameter ($0 < \beta \leq 1$).

$$M = M_{\mathrm{eq}} + (M_0 - M_{\mathrm{eq}})\mathrm{e}^{-\left(\frac{t}{\tau}\right)^{\beta}} \tag{S1}$$

Frequency dependent in-phase (χ') and out-of-phase (χ'') magnetic susceptibility data were collected between 72 and 112 K. The relaxation time for each temperature increment was extracted using the generalized Debye model (eqs S2 and S3) as implemented in *CC-FIT2*.[13] The relaxation times were then tabulated with those from the magnetization decay experiments and fit using CCFIT2.[13]

$$\chi'(\omega) = \chi_S + (\chi_T - \chi_S)\frac{1 + (\omega\tau)^{1-\alpha}\sin(\pi\alpha/2)}{1 + 2(\omega\tau)^{1-\alpha}\sin(\pi\alpha/2) + (\omega\tau)^{2-2\alpha}} \tag{S2}$$

$$\chi''(\omega) = (\chi_T - \chi_S)\frac{(\omega\tau)^{1-\alpha}\cos(\pi\alpha/2)}{1 + 2(\omega\tau)^{1-\alpha}\sin(\pi\alpha/2) + (\omega\tau)^{2-2\alpha}} \tag{S3}$$

1.7. Nuclear Magnetic Resonance Spectroscopy

Proton NMR spectra and proton decoupled ^{13}C NMR spectra were collected at the UC Berkeley College of Chemistry NMR Facility on a Bruker Advance 400 MHz spectrometer operating at 9.4 T under ambient temperature. Samples were sealed under an atmosphere of Ar prior to measurement. Tetrahydrofuran-d8 (99.8%) was purchased from Sigma-Adrich, saturated with argon using three freeze-pump-thaw cycles, and dried for 72 hrs over 3 Å molecular sieves prior to use.

1.8. Powder X-Ray Diffraction

A microcrystalline powder sample of **1** was loaded into a 1.0-mm borosilicate capillary inside a glovebox under an argon atmosphere. The capillary was subsequently flame sealed under Ar prior to measurement. High-resolution synchrotron X-ray powder diffraction data was collected at Beamline 17-BM at the Advanced Photon Source at Argonne National Laboratory. The temperature of the capillary samples was maintained at 298 K during measurement using an Oxford Cryosystems Cryostream 800. Scattered intensity was measured by a PerkinElmer a-Si flat panel detector. The average wavelength of all measurements was 0.45236 Å.

2. Computational Methods

2.1. Density Functional Theory (DFT) Calculations

Geometry optimization of the crystal structure and normal mode calculations were performed in the gas-phase on the anion in **1**·2THF using the Gaussian 09d software,[14] with both PBE and PBE0 density functionals and Grimme's empirical dispersion correction. Dysprosium was substituted for yttrium with an isotopic mass of 162.5 to aid self-consistent field (SCF) convergence, for which the Stuttgart RSC 1997 effective core potential (ECP) basis set was used for the 28 core electrons, with the remaining valence electrons being described by the corresponding valence basis set. The cc-pVTZ basis set was used for boron and carbon atoms, whilst the cc-pVDZ was applied for hydrogen atoms.[14] Geometry optimization and Löwdin population analysis[15] was also performed on the free ligands, as described in the main text, using the Gaussian 09d software employing the 6-31G* basis set on all atoms.[16]

2.2. CASSCF-SO Calculations

The OpenMolcas[17] package was used to perform state-averaged complete active space self-consistent field spin-orbit (CASSCF-SO) calculations on the crystallographic and optimized structures of the anion, where the active space comprised nine 4f electrons in seven 4f orbitals of Dy^{III}. Basis sets were taken from the ANO-RCC library,[18] where the Dy atom had VTZP quality, the five-membered heterocycle was modelled with VDZP quality, with all other atoms in VDZ quality. After CASSCF optimization, the states were mixed by spin-orbit coupling using the RASSI module and the electronic structures projected onto a crystal field Hamiltonian using SINGLE_ANISO.[19]

2.3. Spin Dynamics Calculations

To reduce computational demand for spin dynamics calculations, we limited our CASSCF-SO calculations to the lowest 18 states of sextet multiplicity and used the atomic compact Cholesky decomposition (acCD) approach to fit the two-electron integrals. To check the validity of this approximation, we also calculated the electronic structure of **1-PBE** and **1-PBE0** at a higher level considering 21 sextets along with 244 quartets and 490 doublets in the CASSCF step, and 128 quartets and 130 doublets in the spin-orbit coupling step; we found only a trivial difference between the results (Tables S10–S14). Assuming the harmonic approximation, the maximum displacement along each mode at 100 K was calculated, and the optimized structure was then distorted along the normal mode coordinates. The CASSCF-SO calculations were then performed at four evenly spaced points in the positive and negative direction from the equilibrium position and crystal field parameters extracted. Following the most recently defined method,[20] we employed Bose-Einstein statistics and expanded the crystal field parameters in a Taylor series. Our approach herein differs only in the definition of zero-point displacement (ZPD), herein we define the ZPD as specified in ref. [21] by eq S4, where $\hbar$ is the reduced Planck constant, ω_j is the angular frequency, and μ_j is the reduced mass of mode j.

$$Q_{j,0} = \sqrt{\frac{\hbar}{\omega_j \mu_j}} \tag{S4}$$

2.4. Mode-Weighted Analysis

We use the same methodology described in ref. [20] to decompose total gamma matrices into their mode-weighted components, where $\langle \bar{\bar{H}}_{\mathrm{SP}} \rangle$ is the spin-phonon coupling, $\langle \bar{\bar{Q}} \rangle$ is the vibrational occupancy, $\langle \bar{\bar{\rho}} \rangle$ is the vibrational density of states and $\langle \bar{\bar{n}} \rangle$ is the effective number of modes. This enabled the switching of components between **1**, $[Cp^{iPr5}Dy^{III}Cp^{*}][B(C_6F_5)_4]$,[22] and $[Dy(Cp^{iPr4})_2][B(C_6F_5)_4]$[23] to form fictitious total rate matrices which were diagonalized to give fictitious relaxation times. By comparing the fictitious relaxation times to the experimental relaxation times, we were able to elucidate the factors that most substantially contribute to the differences in the relaxation between **1** and $[Cp^{iPr5}Dy^{III}Cp^{*}][B(C_6F_5)_4]$ or $[Dy(Cp^{iPr4})_2][B(C_6F_5)_4]$.

3. Figures and Tables

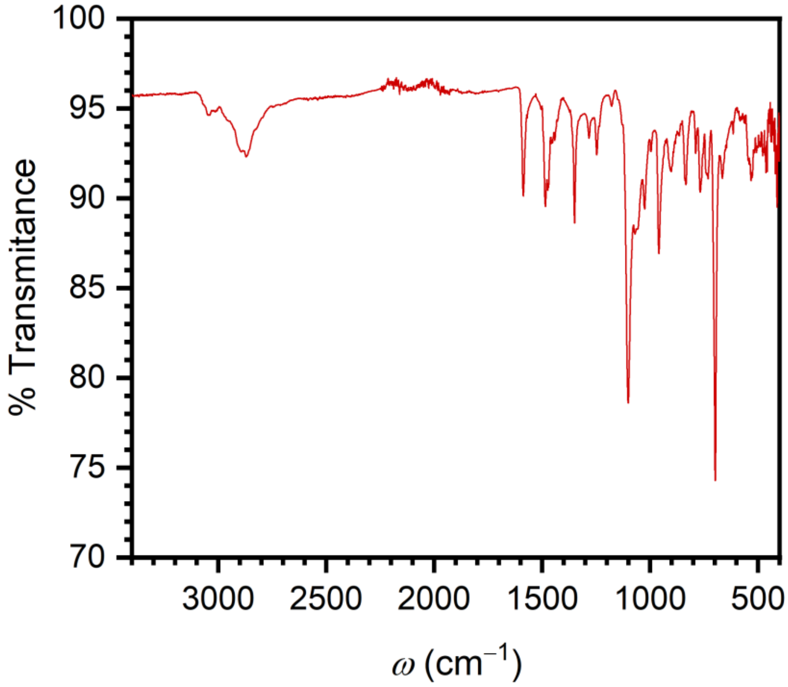

Figure S1. Fourier-transform infrared spectrum of **1**.

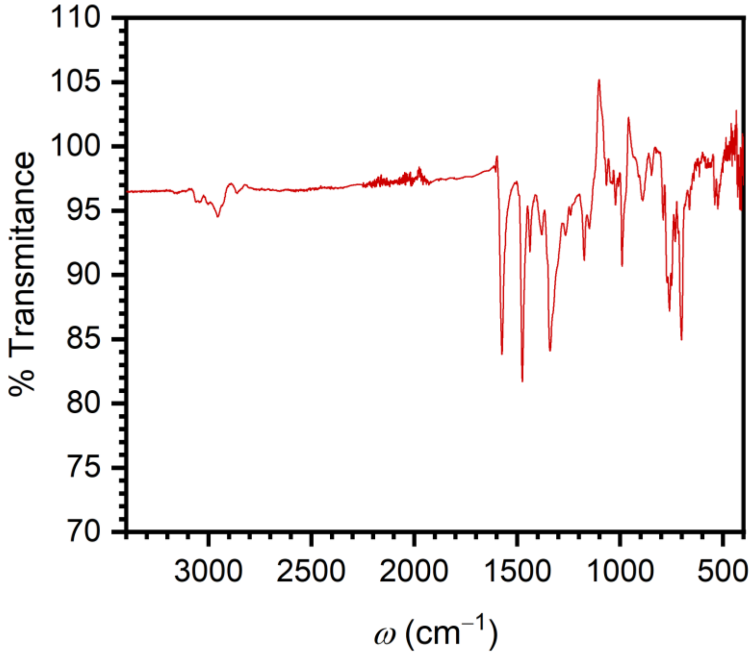

Figure S2. Fourier transform infrared spectrum of putative **1**·2THF

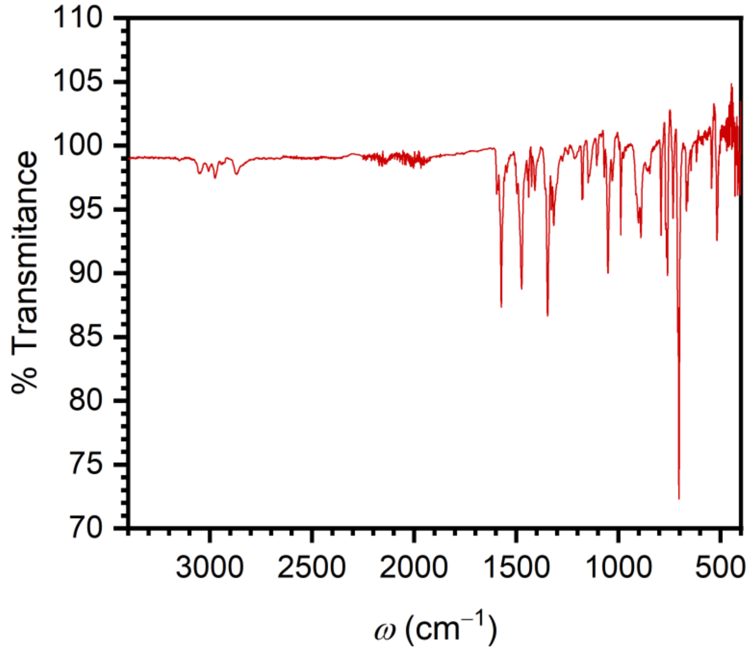

Figure S3. Fourier-transform infrared spectrum of $K_2BC_4Ph_5$.

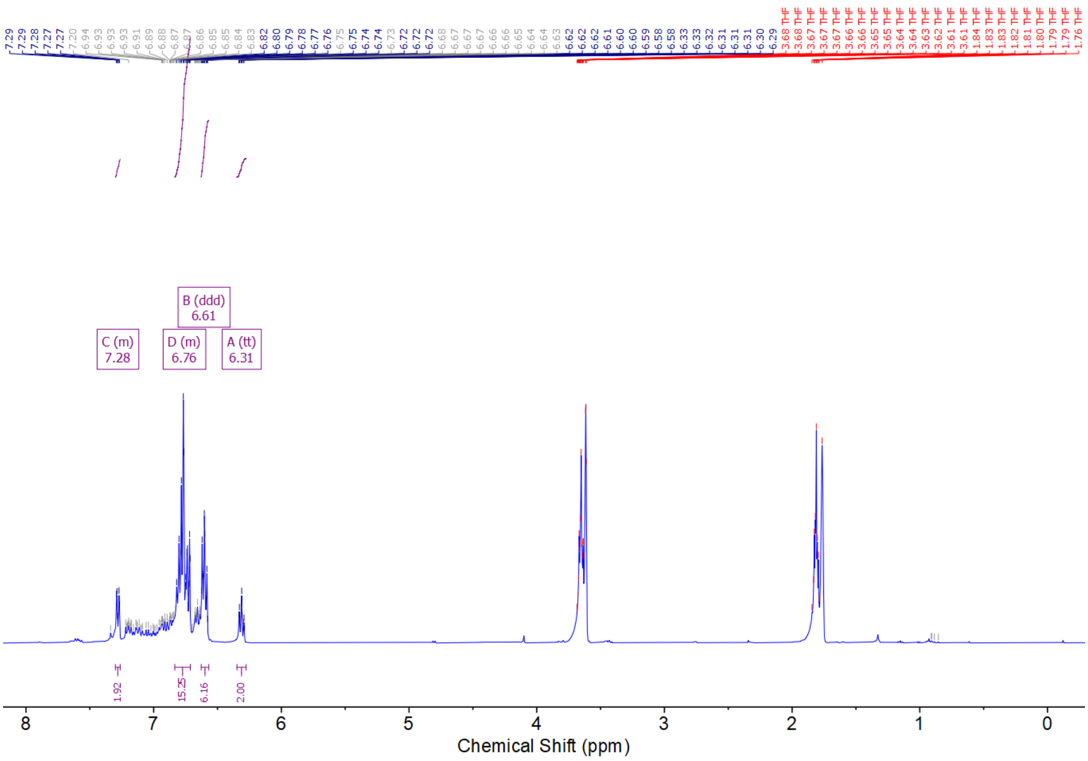

Figure S4. ^{1}H NMR Spectrum of $K_2BC_4Ph_5$. ^{1}H NMR (400 MHz, THF) δ 7.30 – 7.26 (m, 2H), 6.83 – 6.71 (m, 15H), 6.61 (ddd, J = 10.2, 5.4, 2.0 Hz, 6H), 6.31 (tt, J = 7.1, 1.4 Hz, 2H).

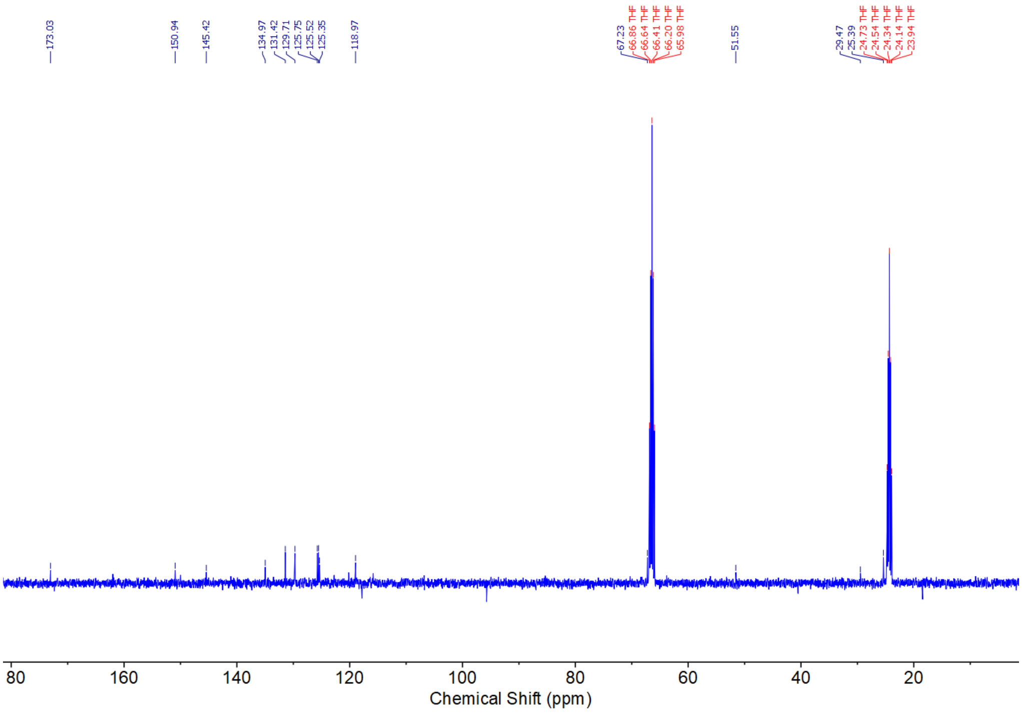

Figure S5. ^{13}C NMR spectrum of $K_2BC_4Ph_5$. ^{13}C NMR (101 MHz, THF) δ 173.03, 150.94, 145.42, 134.97, 131.42, 129.71, 125.75, 125.52, 125.35, 118.97, 67.23, 51.55, 29.47, 25.39.

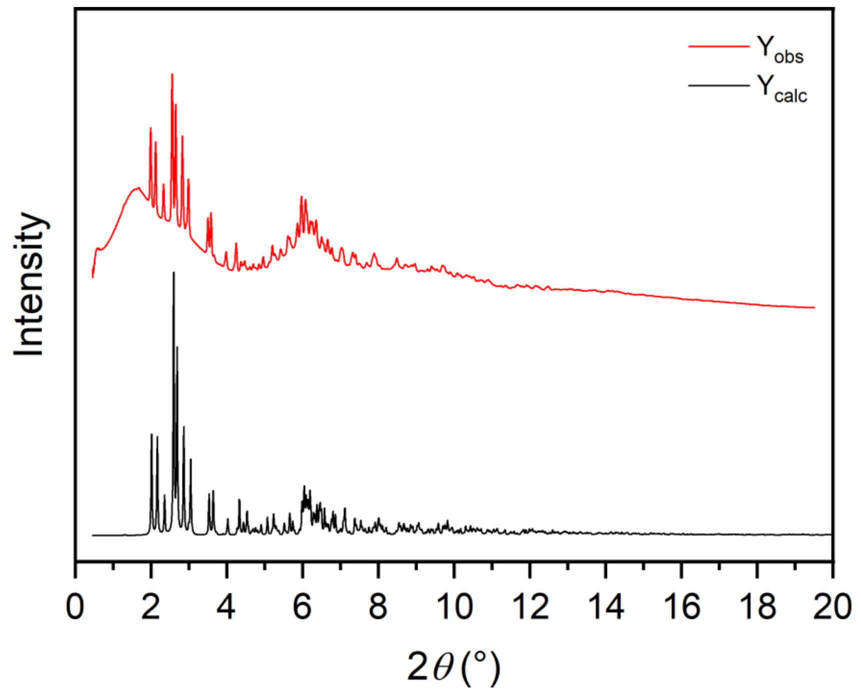

Figure S6. Comparison of powder x-ray diffraction pattern obtained for mechanically crushed crystals of **1** (red trace) with the simulated diffraction pattern based on the single-crystal structure of **1**·2THF (black trace). The sharp peaks in both patterns overlap, suggesting that the bulk structure of desolvated **1** and **1**·2THF are the same. The broadness, particularly at low angles, in the experimental pattern for **1** is likely background scattering from the borosilicate sample capillary.

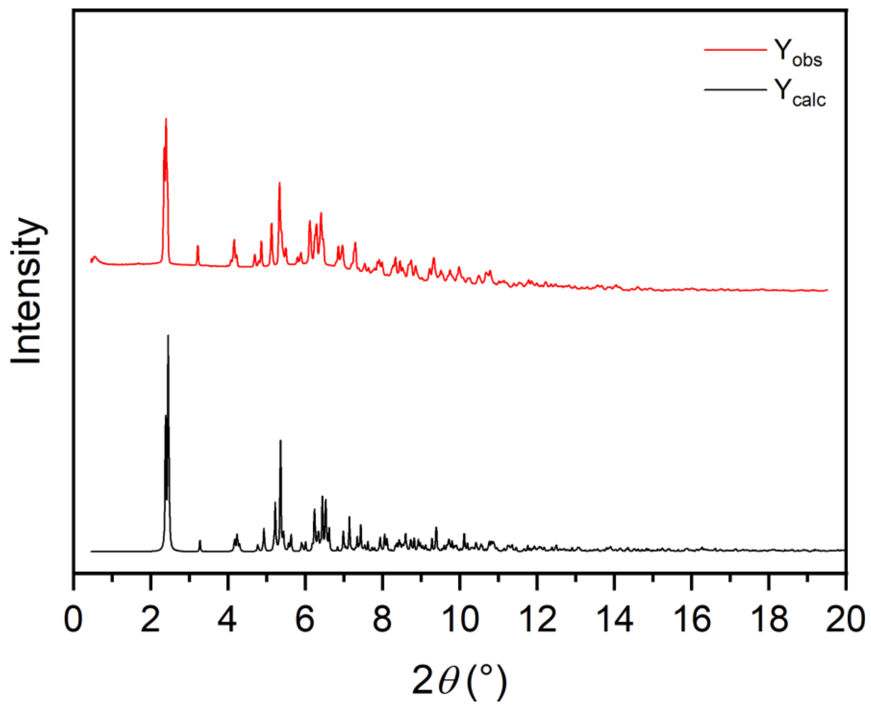

Figure S7. Comparison of powder x-ray diffraction pattern obtained for $K_2BC_4Ph_5$ (red trace) with a simulated pattern derived from CCDC:701219 (black trace).[24]

Table S1. Unit Cell Parameters and Refinement Metrics for **1**·2THF.

Empirical formula	$C_{88}H_{90}B_2Dy_{0.97}KO_8$
Formula weight	1494.75
Temperature/K	100
Crystal system	triclinic
Space group	P-1
a/Å	12.547(5)
b/Å	15.210(6)
c/Å	20.620(8)
α/°	96.799(4)
β/°	100.948(4)
γ/°	101.827(4)
Volume/Å^3	3731(3)
Z	2
ρ_{calc}g/cm^3	1.331
μ/mm^{-1}	1.155
F(000)	1551.0
Crystal size/mm^3	0.05 × 0.05 × 0.04
Radiation	synchrotron (λ = 0.7288)
2Θ range for data collection/°	2.844 to 54.194
Index ranges	−15 ≤ h ≤ 15, −19 ≤ k ≤ 18, −25 ≤ l ≤ 25
Reflections collected	64045
Independent reflections	15231 [R_{int} = 0.0656, R_{sigma} = 0.0580]
Data/restraints/parameters	15231/235/1069
Goodness-of-fit on F^2	1.031
Final R indexes [I>=2σ (I)]	R_1 = 0.0572, wR_2 = 0.1582
Final R indexes [all data]	R_1 = 0.0721, wR_2 = 0.1701

Table S2. Selected Distances and Angles for **1**·2THF.

Anion Index	Dy Site	Occupancy	Dy–centroid (Å)	centroid–Dy–centroid angle (°)
1	Dy1A	0.35523	2.3491(13)<br>2.2641(10)	155.99(13)
	Dy1B	0.12429	2.334(3)<br>2.275(2)	156.5(4)
2	Dy2A	0.46247	2.2118(8)<br>2.4187(8)	156.18(2)
	Dy2B	0.03418	2.392(6)<br>2.266(6)	153.2(3)
Average			2.33 ± 0.09	156 ± 1

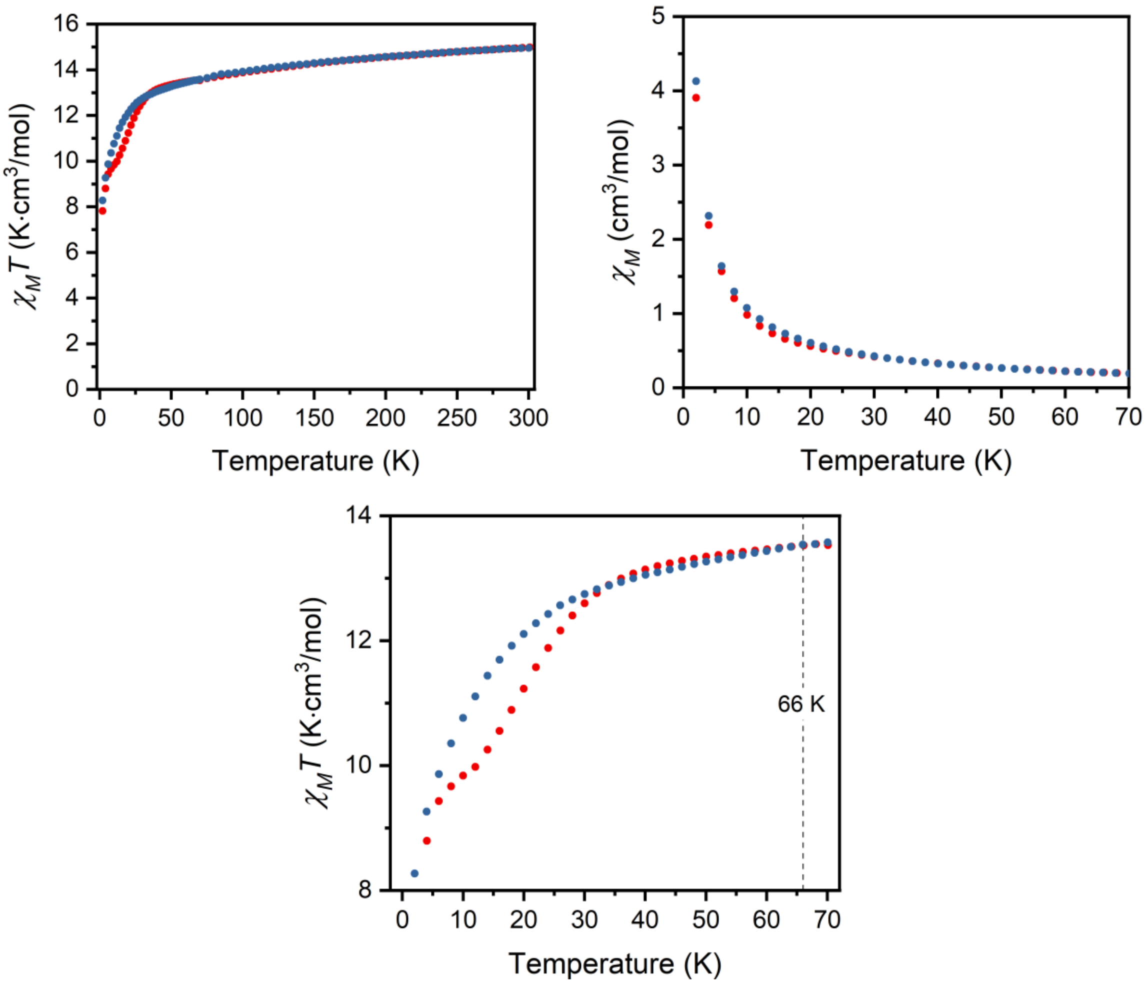

Figure S8. (Upper left) Temperature dependence of the molar magnetic susceptibility times temperature ($\chi_M T$) for a sample of one **1** cooled under zero dc field (red symbols) and under a field of 0.1 T (blue symbols). All data were collected under H_{dc} = 0.1 T. (Upper right) Temperature dependence of the molar magnetic susceptibility (χ_M) for a sample of one **1** cooled under zero dc field (red symbols) and under a field of 0.1 T (blue symbols). All data were collected under H_{dc} = 0.1 T. (Lower) Expanded view of the data in the upper plot from 2 to 70 K, showing T_{irrev} at 66 K.

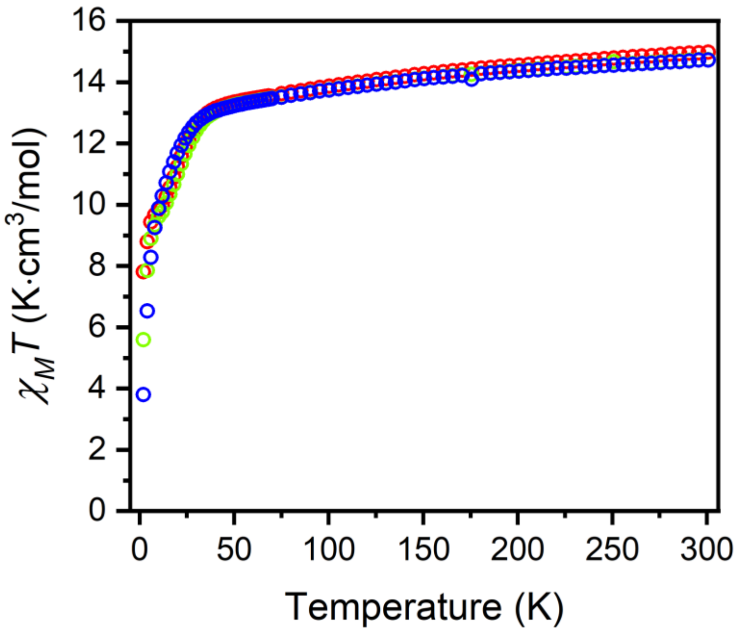

Figure S9. Temperature dependence of the molar magnetic susceptibility times temperature ($\chi_M T$) for a sample of one **1** cooled under zero dc field. Blue, green, and red symbols correspond to data collected under dc fields of 1, 0.5, and 0.1 T, respectively.

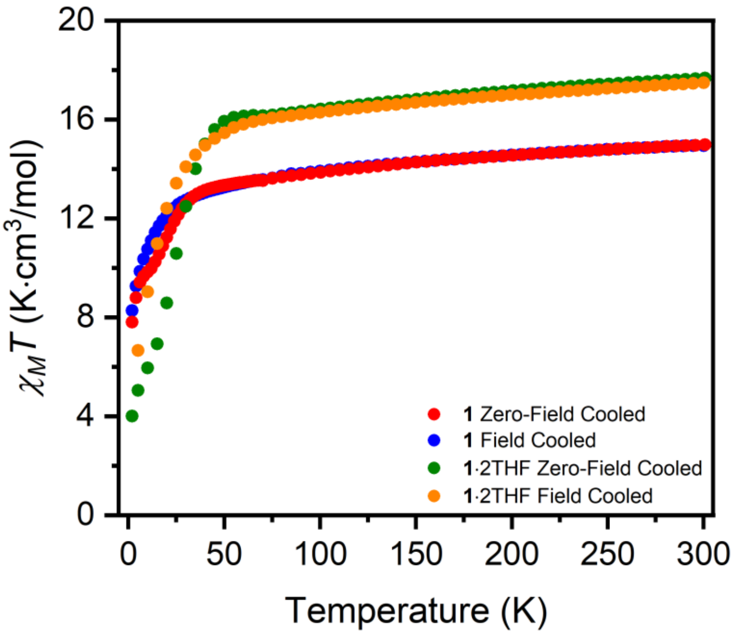

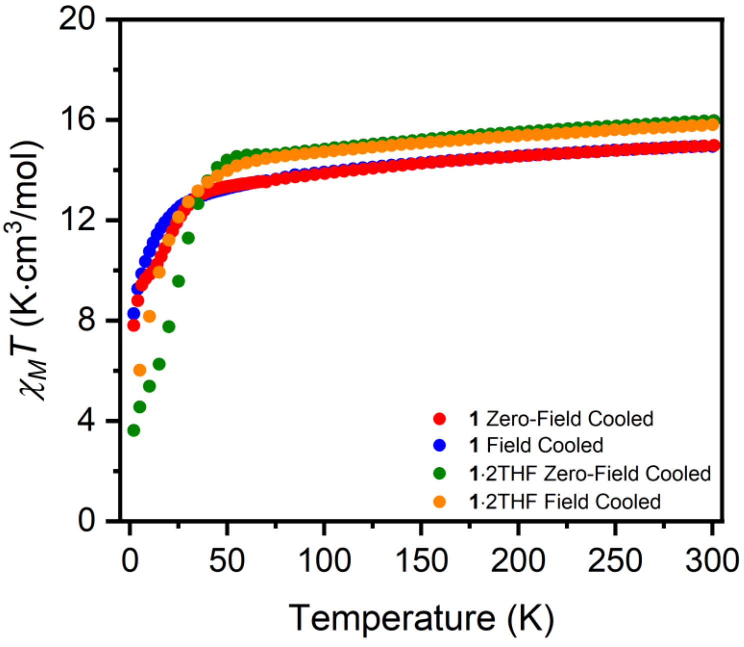

Figure S10. (Upper) Temperature dependence of the molar magnetic susceptibility times temperature ($\chi_M T$) for a sample of purported **1**·2THF cooled under zero dc field (green symbols) and under a field of 0.1 T (orange symbols). Data were worked up using the measured sample mass of 12.6 mg and using the molecular mass of 1498.9 g/mol for **1**·2THF. It is clear that the magnitude of the susceptibility is much higher than that measured for **1** at room temperature (red symbols represent zero-field cooled data and blue symbols represent field-cooled data, both measured under 0.1 T applied field) and much higher than predicted for free Dy^{3+}, 14.17 emu·K/mol, suggesting the molar mass used does not reflect the actual composition of the sample. (Lower) Adjusted temperature dependence of the molar magnetic susceptibility times temperature ($\chi_M T$) for the sample of purported **1**·2THF, where the sample molar mass was set to that of desolvated **1**, 1354.7 g/mol, which brings the magnitude of $\chi_M T$ at 300 K in into near agreement with that obtained for the fully desolvated sample **1**. This suggests that the sample of **1**·2THF used to prepare the second magnetic sample desolvates at least partially.

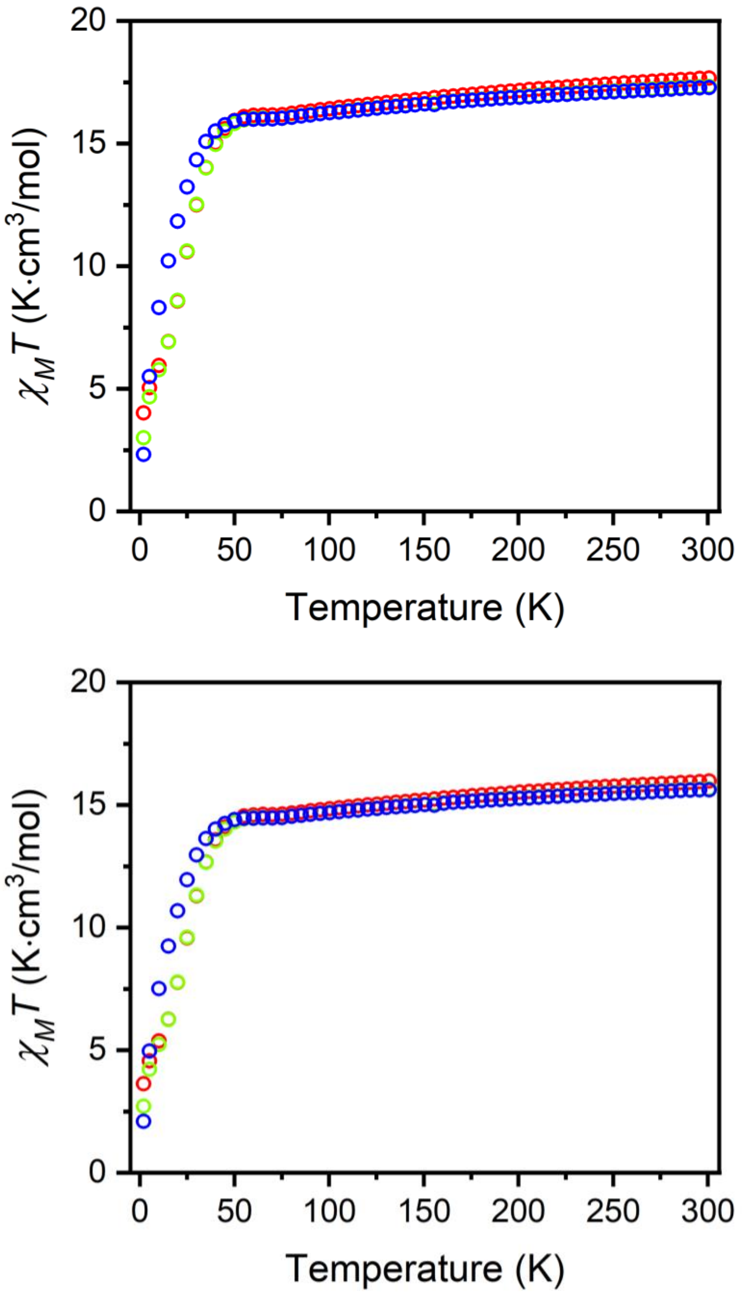

Figure S11. (Upper) Temperature dependence of the molar magnetic susceptibility times temperature ($\chi_M T$) for a sample of putative **1**·2THF cooled under zero dc field. Blue, green, and red symbols correspond to data collected under dc fields of 1, 0.5, and 0.1 T, respectively. Data were worked up using the measured sample mass of 12.6 mg and using the molecular mass of 1498.9 g/mol for **1**·2THF. It is clear that the magnitude of the susceptibility is much higher than that measured for **1** at room temperature (see Figure S9) and also much higher than predicted for free Dy^{3+} (14.17 emu·K/mol), indicating the molar mass used does not reflect the composition of the sample. (Lower) Adjustment of the data shown in the upper panel using a molar mass of 1354.7 g/mol, corresponding to desolvated **1**, brings the $\chi_M T$ magnitudes into near agreement with those collected for **1**, suggesting that the **1**·2THF used to prepare the second magnetic sample is at least partially desolvated.

Table S3. Tabulated Decay Fit Parameters for **1**. Estimated uncertainties in τ at the 1σ level were calculated with $\tau_{\pm} = \tau \exp\{\pm \frac{1.64 \tan[\frac{\pi}{2}(1-\beta)]}{(1-\beta)^{0.141}}\}$ derived in Ref. 22 where τ and β are the fitted values from the stretched exponential function, and $\tau_{\pm}$ are the limits of the 1σ uncertainties.

T **(K)**	**τ (s)**	***β***	**τ_+**	**τ_-**
2	1277	0.774	1425	673
3	1086	0.710	1739	669
4	1093	0.711	1741	671
5	1094	0.712	1733	671
6	1079	0.716	1673	656
7	1046	0.713	1648	640
8	1024	0.726	1503	609
9	966	0.730	1387	569
10	926	0.741	1250	532
12	826	0.754	1036	460
14	729	0.760	883	399
16	647	0.757	797	357
18	583	0.754	731	324
20	526	0.747	686	298
22	480	0.739	655	277
24	443	0.733	625	259
28	383	0.731	547	225
32	338	0.733	477	198
36	297	0.738	408	172
40	244	0.760	296	134
44	244	0.760	296	134
48	227	0.778	247	118
52	201	0.776	222	105
56	185	0.813	162	86
60	157	0.791	158	79
64	131	0.862	80	50

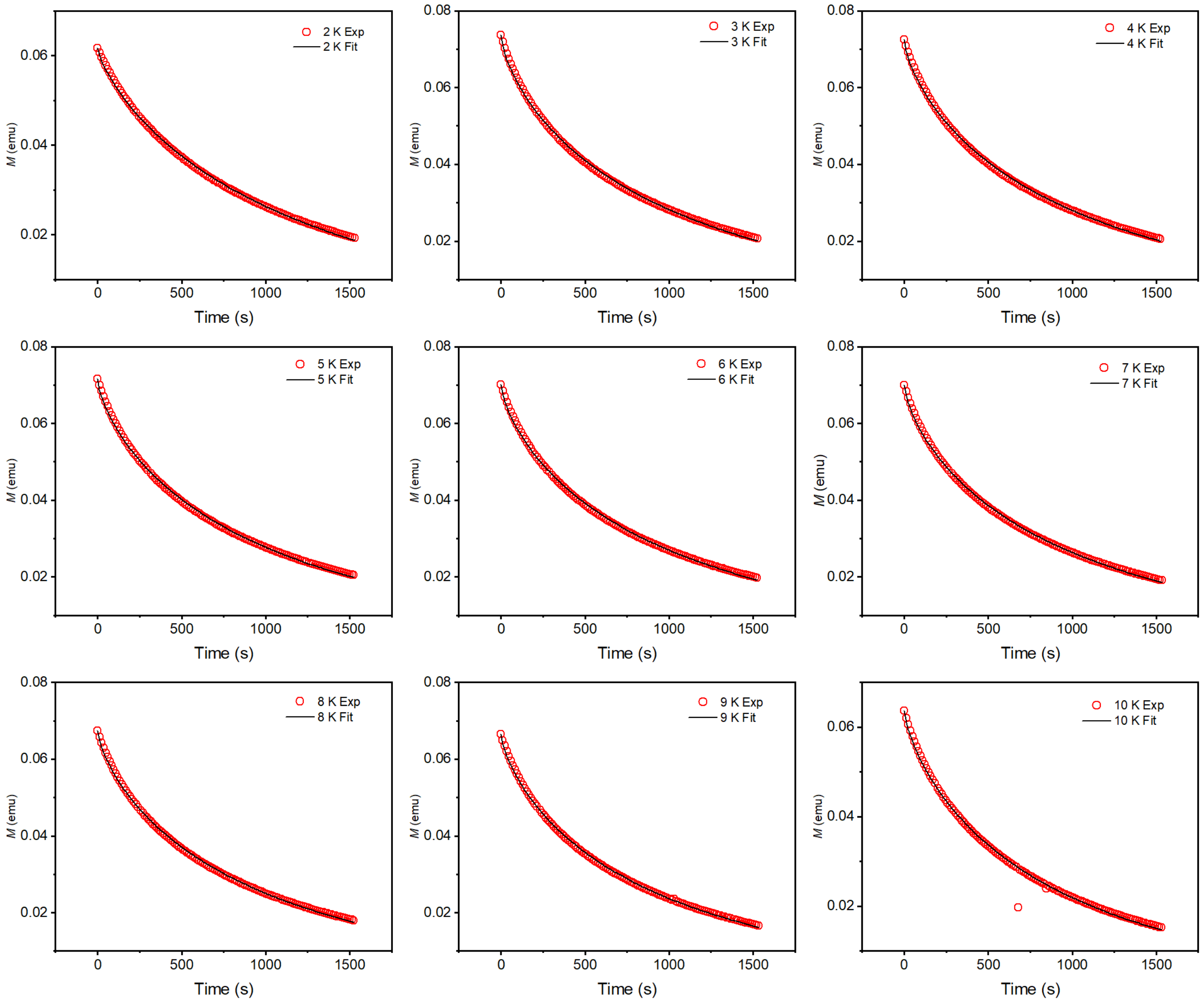

Figure S12. Normalized magnetization decay plots for **1** collected at the indicated temperatures. Circles indicate data points and black lines are fits using eq S1. The sample was initially magnetized under an applied magnetic field of 7 T for 5 min prior to data collection.

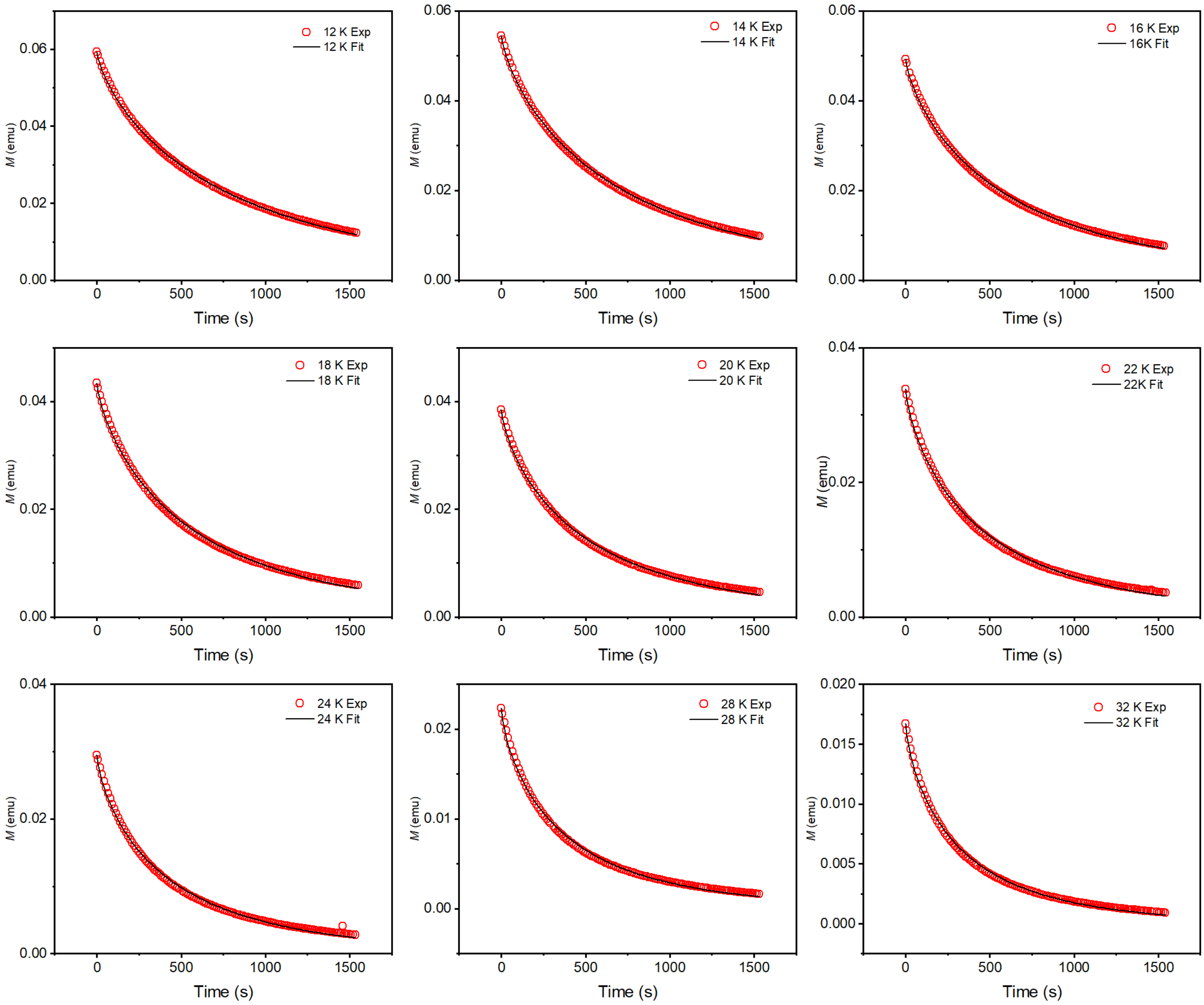

Figure S13. Normalized magnetization decay plots for **1** collected at the indicated temperatures. Circles indicate data points and black lines are fits using eq S1. The sample was initially magnetized under an applied magnetic field of 7 T for 5 min prior to data collection.

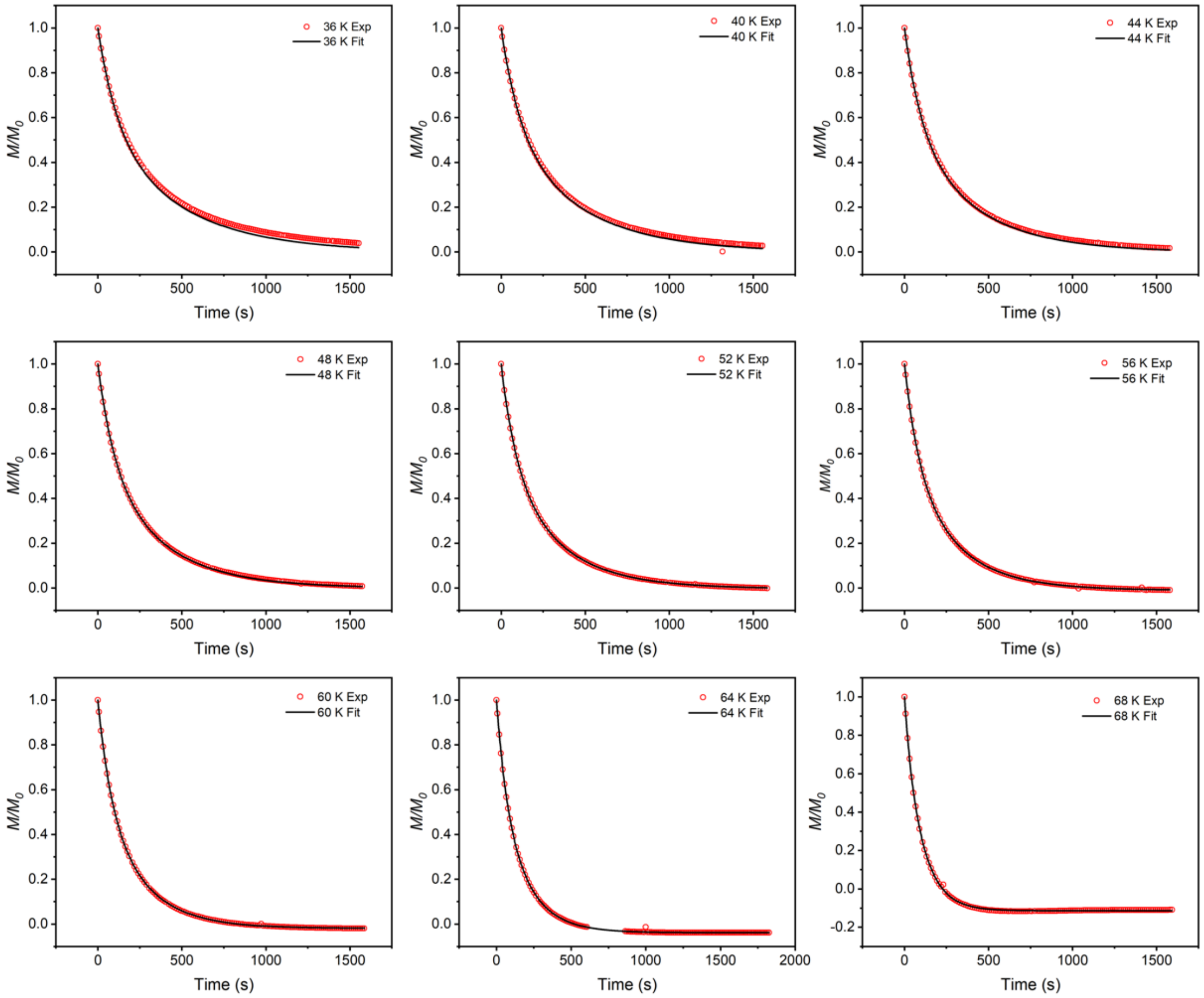

Figure S14: Normalized magnetization decay plots for **1** collected at the indicated temperatures. Circles indicate data points and black lines are fits using eq S1. The sample was initially magnetized under an applied magnetic field of 7 T for 5 min prior to data collection.

Table S4. Tabulated Decay Fit Parameters obtained for a magnetic sample prepared from crystals of **1**·2THF (see Section 1.6). Data are intended to verify reproducibility of data collected for **1** only. Estimated uncertainties in τ at the 1σ level were calculated with $\tau_{\pm} = \tau \exp\{\pm \frac{1.64 \tan[\frac{\pi}{2}(1-\beta)]}{(1-\beta)^{0.141}}\}$ derived in Ref. 22 where τ and β are the fitted values from the stretched exponential function, and $\tau_{\pm}$ are the limits of the 1σ uncertainties.

T **(K)**	**τ (s)**	***β***	τ_{+}	τ_{-}
2	1048	0.688	1884	674
4	988	0.694	1722	628
6	951	0.690	1691	609
8	944	0.698	1618	597
10	883	0.708	1430	546
12	823	0.752	1046	461
14	742	0.721	1119	446
16	693	0.727	1012	412
18	635	0.729	916	375
20	575	0.725	848	343
22	540	0.728	788	320
24	503	0.728	728	297
28	444	0.733	625	260
32	393	0.738	539	227
36	345	0.740	468	198
40	315	0.750	403	177
44	286	0.760	346	157
48	260	0.770	297	139
52	234	0.777	256	122
56	228	0.879	120	79
60	148	0.797	144	73
64	147	0.877	79	51
68	105	0.875	57	37

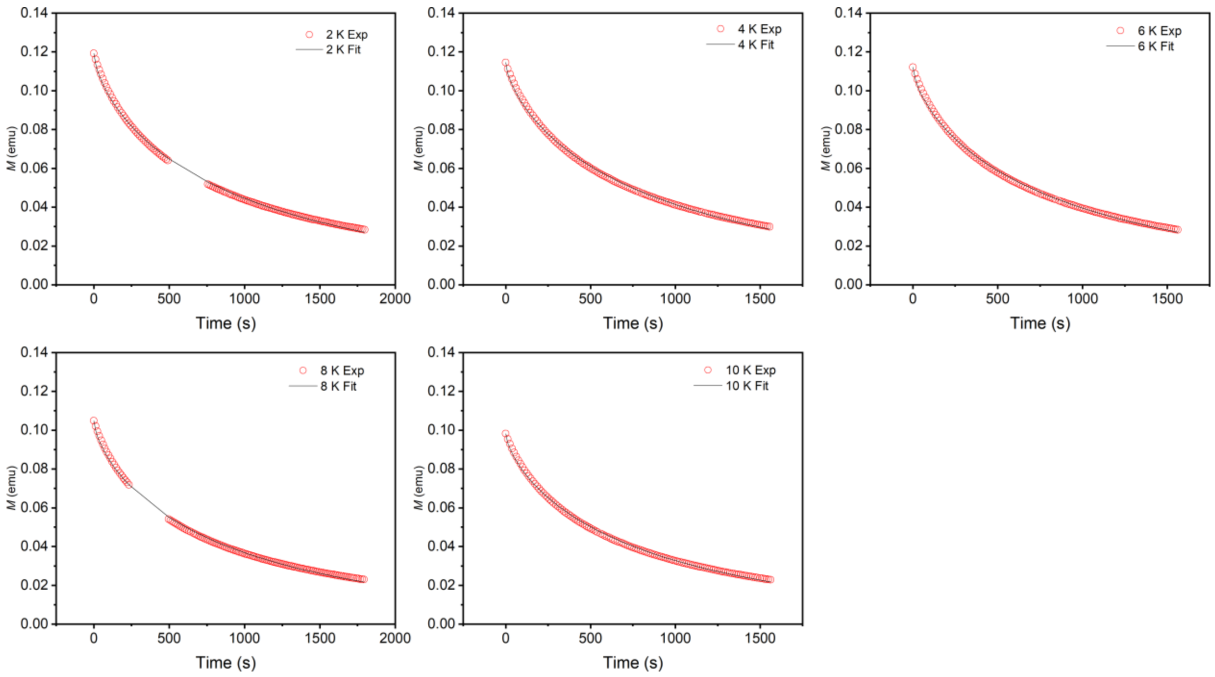

Figure S15. Normalized magnetization decay plots obtained for a magnetic sample prepared from crystals of **1**·2THF (see Section 1.6). Data are shown only to verify reproducibility of data collected for **1**, given the uncertainty in the solvation state of the compound. Circles indicate data points and black lines are fits using eq S1. The sample was initially magnetized under an applied magnetic field of 7 T for 5 min prior to data collection.

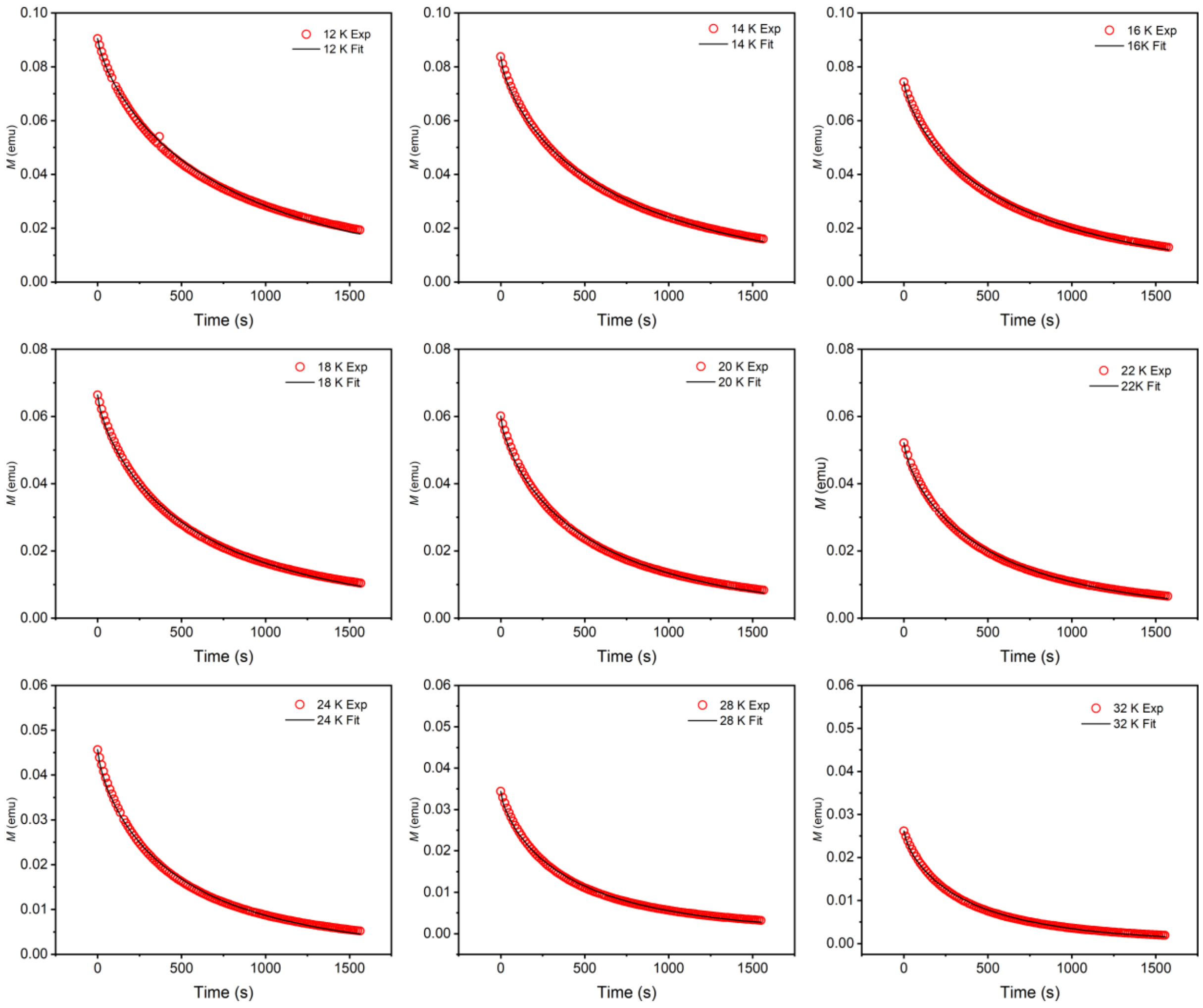

Figure S16. Normalized magnetization decay plots obtained for a magnetic sample prepared from **1**·2THF. Data are shown only to verify reproducibility of data collected for **1**, due to the uncertainty in the solvation state of the compound. Circles indicate data points and black lines are fits using eq S1. The sample was initially magnetized under an applied magnetic field of 7 T for 5 min prior to data collection.

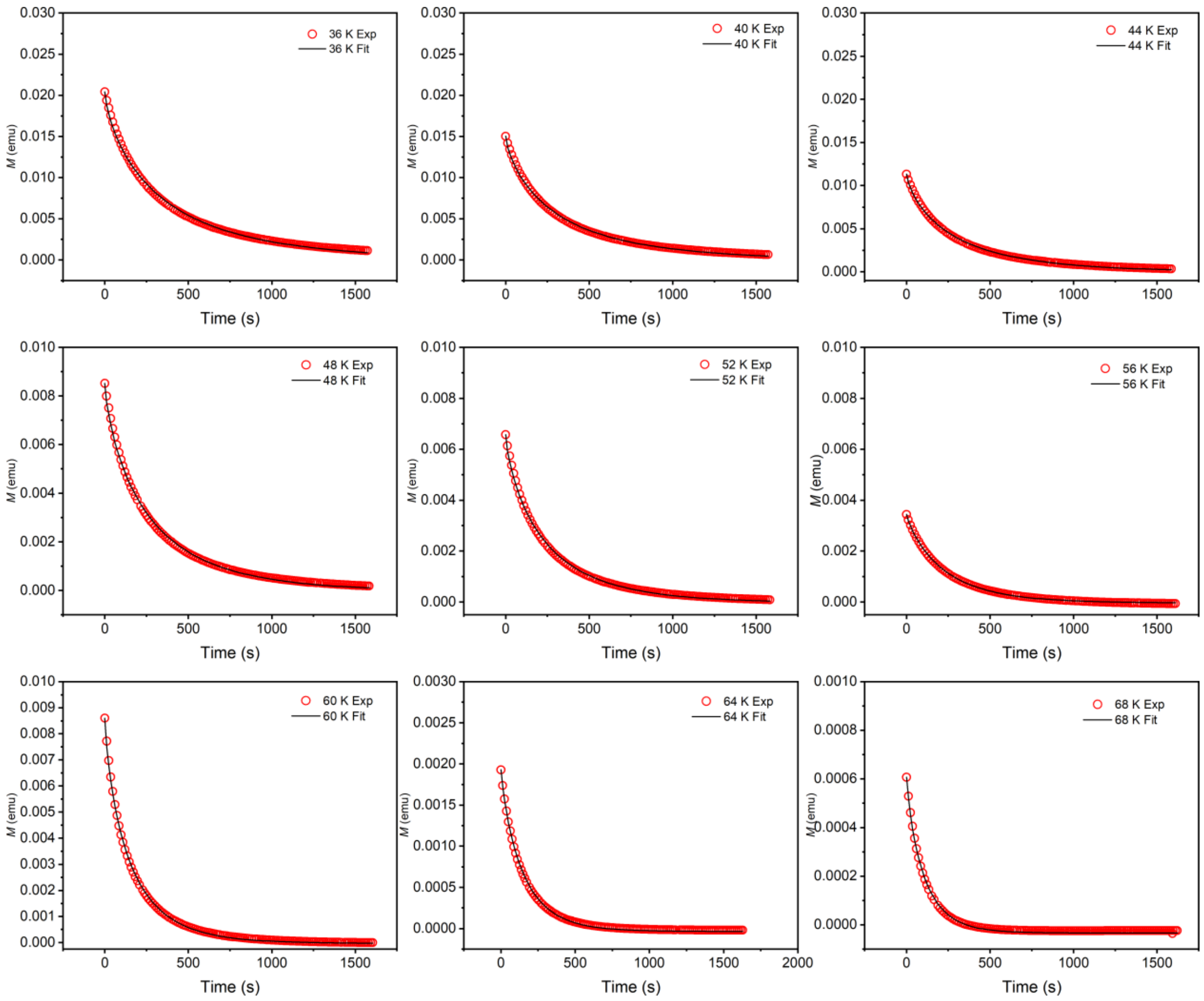

Figure S17. Normalized magnetization decay plots obtained for a magnetic sample prepared from **1**·2THF. Data are only intended to verify reproducibility of data collected for **1**, due to the uncertainty in the solvation state of the compound. Circles indicate data points and black lines are fits using eq S1. The sample was initially magnetized under an applied magnetic field of 7 T for 5 min prior to data collection.

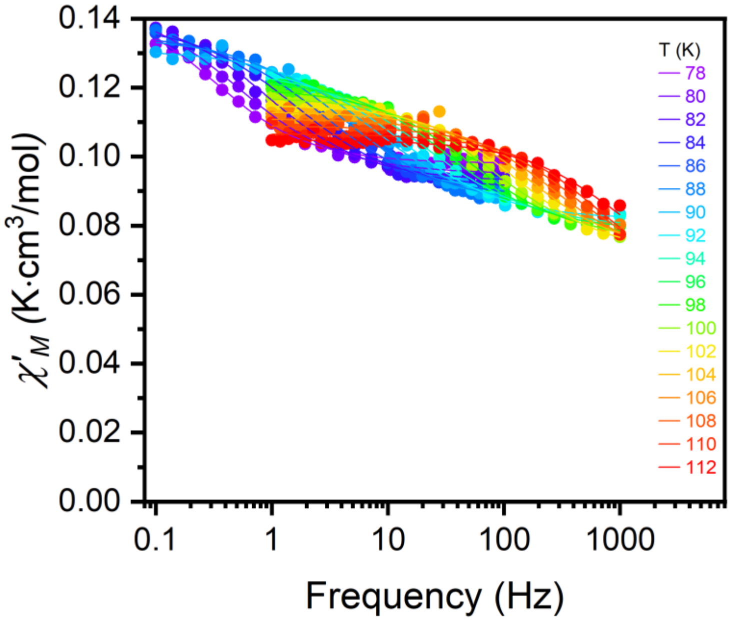

Figure S18. Molar in-phase ac magnetic susceptibility versus frequency data for **1**, collected under zero dc field. Symbols represent experimental data and lines are fits to the generalized Debye model (eqs S2 and S3). The susceptibility does not go to zero at the highest frequencies, suggesting that there is a second, faster relaxation process occurring outside of the measured frequency range.

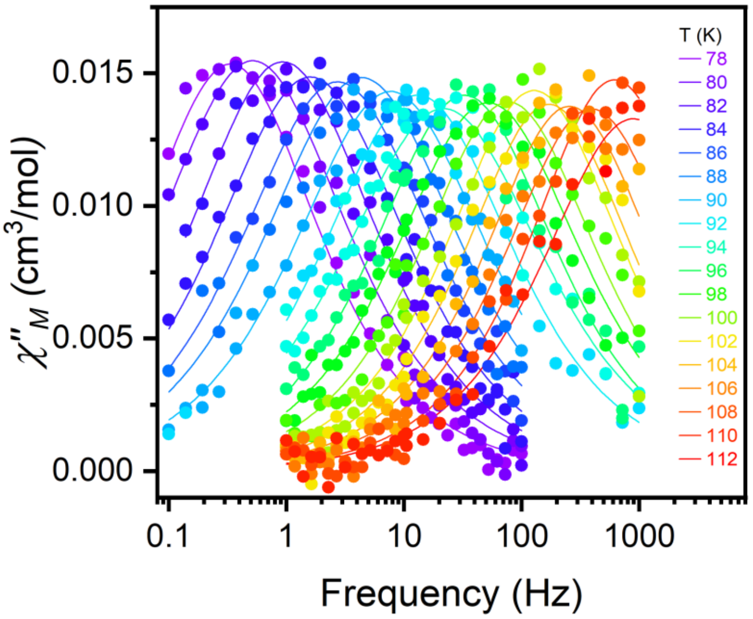

Figure S19. Molar out-of-phase ac magnetic susceptibility versus frequency data for **1**, collected under zero dc field. Symbols represent experimental data and lines are fits to the generalized Debye model (eqs S2 and S3).

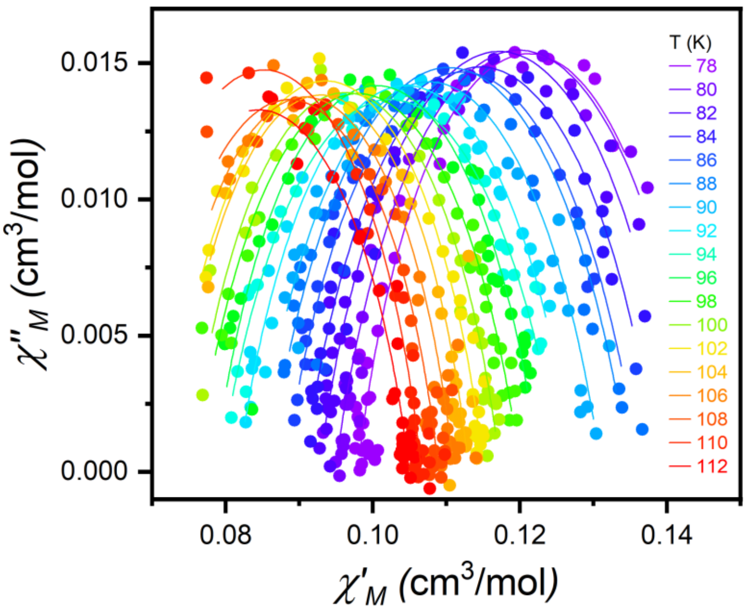

Figure S20. Cole-Cole plots for **1** generated from zero-field ac susceptibility data. Symbols represent experimental data and lines are fits to the generalized Debye model (eqs S2 and S3).

Table S5. Ac susceptibility fit parameters for **1**. All χ_S and χ values are reported in emu/mol.

T (K)	τ (s)	τ Error (s)	χ_S	χ_S Error	χ_T	χ_T Error	α	α error
78	0.48	0.03	0.0979	0.0002	0.144	0.001	0.26	0.02
80	0.31	0.02	0.0948	0.0003	0.145	0.001	0.29	0.02
82	0.171	0.008	0.0926	0.0003	0.1417	0.0009	0.29	0.02
84	0.099	0.004	0.0905	0.0004	0.1388	0.0007	0.30	0.02
86	0.058	0.002	0.0880	0.0004	0.1362	0.0007	0.30	0.02
88	0.037	0.002	0.0857	0.0007	0.1349	0.0007	0.31	0.02
90	0.0207	0.0008	0.0841	0.0006	0.1311	0.0005	0.30	0.02
92	0.0121	0.0003	0.0816	0.0003	0.1274	0.0005	0.29	0.01
94	0.0074	0.0003	0.0791	0.0005	0.1255	0.0005	0.32	0.02
96	0.0049	0.0002	0.0784	0.0005	0.1227	0.0004	0.27	0.01
98	0.0031	0.0001	0.0759	0.0005	0.1201	0.0003	0.28	0.01
100	0.0021	0.0001	0.0751	0.0007	0.1173	0.0003	0.26	0.02
102	0.0012	0.0000	0.0720	0.0006	0.1151	0.0002	0.25	0.01
104	0.00093	0.00005	0.073	0.001	0.1131	0.0003	0.24	0.02
106	0.00062	0.00005	0.071	0.001	0.1110	0.0003	0.24	0.03
108	0.00041	0.00003	0.068	0.002	0.1090	0.0003	0.25	0.02
110	0.00026	0.00003	0.064	0.003	0.1066	0.0003	0.23	0.03
112	0.00018	0.00003	0.064	0.003	0.1050	0.0002	0.27	0.03

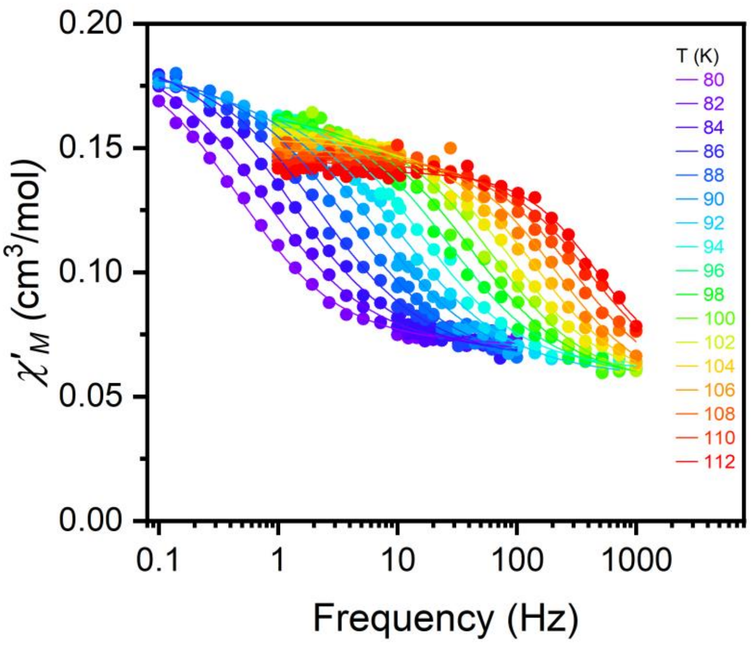

Figure S21. Molar in-phase ac magnetic susceptibility versus frequency data obtained under zero applied field for a magnetic sample prepared from **1**·2THF. Based on dc magnetic susceptibility measured for this sample (Figures S10 and S11), it is likely at least partially desolvated, and so data serve only to support reproducibility of data collected for **1**. Symbols represent experimental data and lines are fits to the generalized Debye model (eqs S2 and S3). Interestingly, the in-phase ac susceptibility for this sample approaches zero more closely than the same data for **1** (see Figure S18). This is the only readily apparent difference between the data obtained for the two samples.

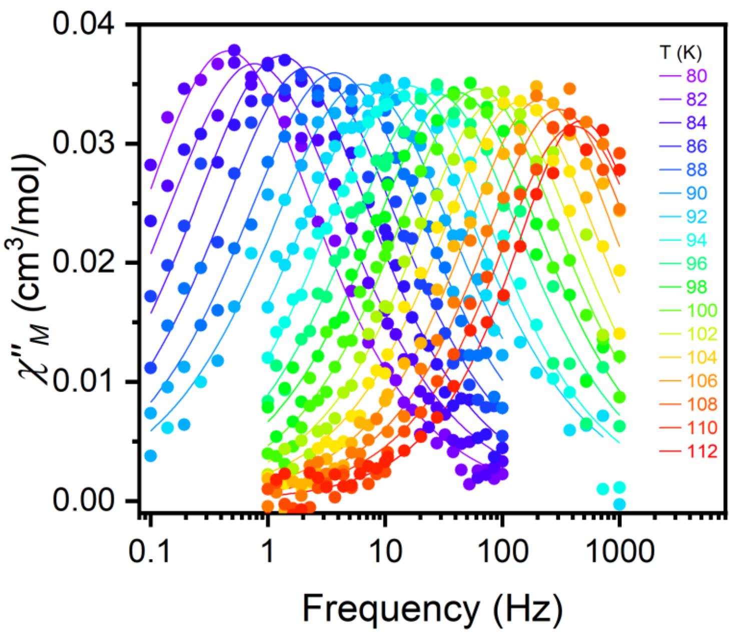

Figure S22. Molar out-of-phase ac magnetic susceptibility versus frequency data collected under zero applied field for a magnetic sample prepared from **1**·2THF. Based on dc magnetic susceptibility measured for this sample (Figures S10 and S11), it is likely partially desolvated, and so data serve only to support reproducibility of data collected for **1**. Symbols represent experimental data and lines are fits to the generalized Debye model (eqs S2 and S3).

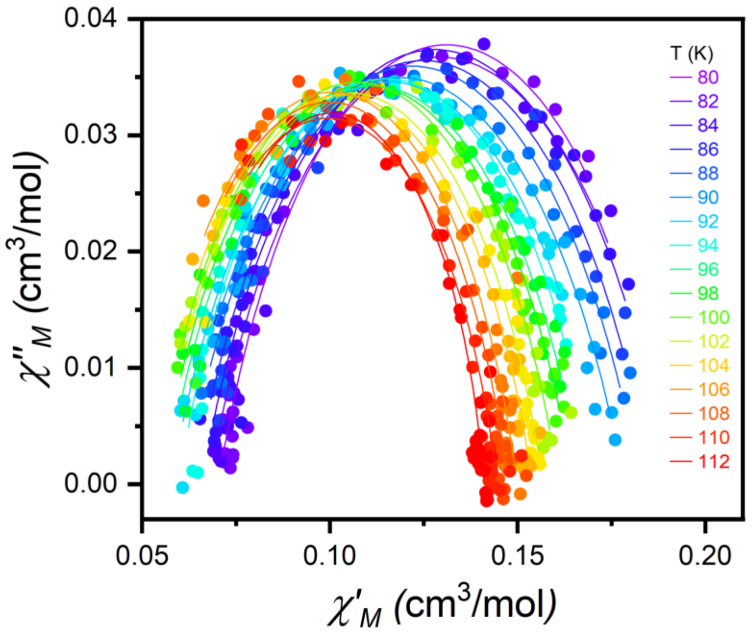

Figure S23. Cole-Cole plot of out-of-phase susceptibility versus in-phase susceptibility data collected under zero applied field for a magnetic sample prepared from **1**·2THF. Based on dc magnetic susceptibility measured for this sample (Figures S10 and S11), it is likely at least partially desolvated, and so data serve only to support reproducibility of data collected for **1**. Symbols represent experimental data and lines are fits to the generalized Debye model (eqs S2 and S3).

Table S6. Ac susceptibility fit parameters for a magnetic sample prepared from **1**·2THF, obtained from fits to data unadjusted for sample composition (see Figures S10, S11, and Section 1.6), which should affect only the magnitude of χ_S and χ_T (reported in emu/mol), and not the relaxation times or α values. These data are intended to verify reproducibility of data collected for **1**.

T (K)	τ (s)	τ Error (s)	χ_S	χ_S Error	χ_T	χ_T Error	α	α error
80	0.35	0.01	0.0702	0.0005	0.192	0.002	0.29	0.01
82	0.208	0.009	0.0676	0.0007	0.191	0.002	0.32	0.01
84	0.123	0.004	0.0658	0.0007	0.190	0.001	0.31	0.01
86	0.072	0.002	0.0638	0.0007	0.186	0.001	0.31	0.01
88	0.043	0.001	0.0614	0.0009	0.183	0.001	0.32	0.01
90	0.0252	0.0008	0.060	0.001	0.178	0.001	0.32	0.01
92	0.0151	0.0003	0.0575	0.0007	0.174	0.001	0.32	0.01
94	0.0100	0.0003	0.0596	0.0009	0.170	0.001	0.28	0.01
96	0.00597	0.0002	0.056	0.001	0.1672	0.0009	0.30	0.01
98	0.00391	0.0001	0.055	0.001	0.1647	0.0009	0.29	0.01
100	0.00256	0.00008	0.053	0.001	0.1609	0.0007	0.27	0.01
102	0.00178	0.00005	0.054	0.001	0.1567	0.0005	0.26	0.01
104	0.00115	0.00004	0.052	0.001	0.1539	0.0004	0.26	0.01
106	0.00075	0.00004	0.051	0.002	0.1498	0.0006	0.24	0.02
108	0.00052	0.00003	0.052	0.003	0.1474	0.0006	0.23	0.02
110	0.00039	0.00003	0.054	0.003	0.1446	0.0006	0.22	0.02
112	0.00034	0.00002	0.060	0.003	0.1410	0.0004	0.15	0.02

Table S7. Arrhenius fit parameters for a magnetic sample prepared from **1**·2THF, obtained from fits to data unadjusted for sample composition (see Figures S10, S11, and Section 1.6), which should affect only the magnitude of χ_S and χ_T (reported in emu/mol), and not the relaxation times or α values. These data are reported to verify reproducibility of data collected for **1**.

U_{eff} (cm^{1})	τ_0 (s)	C (s^{-1}·K^{n})	n	τ_{QTM} (s)
1600(100)	$10^{-12.6(8)}$	$10^{-5(2)}$	1(1)	$10^{3.1(3)}$

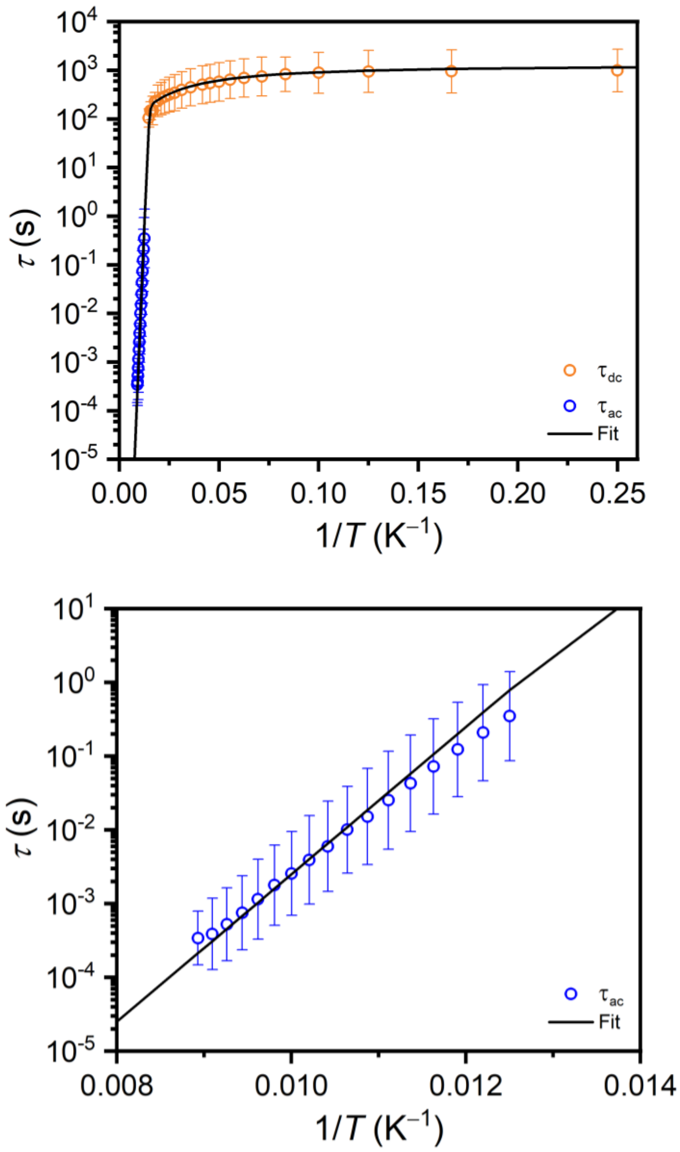

Figure S24. (Upper) Arrhenius plot of average relaxation times obtained for a magnetic sample prepared from **1**·2THF as described above (with 1σ uncertainties). These data are reported to verify reproducibility of data collected for **1**. Orange circles correspond to relaxation times from magnetization decay, blue circles correspond to relaxation times from ac susceptibility measurements. (Lower) Expanded view of the ac region in the upper plot. Fit curve generated using fitted parameters in Table S7 with Eq 1 from the main text.

Table S8. Cartesian coordinates of the PBE and PBE0 optimized structures of the $[Dy(BC_4Ph_5)_2]^-$ anion in **1**·2THF.

	**1-PBE**			**1-PBE0**		
Dy	-0.34436	-0.23783	-0.02990	-0.33466	-0.19237	-0.04002
C	1.23979	2.41515	2.08639	1.28871	2.37535	2.08167
C	0.98129	-0.15792	2.21921	0.93754	-0.16858	2.21905
C	-2.89504	-0.93085	2.23033	-2.94438	-0.80955	2.20391
C	-0.09890	-1.11564	2.37760	-0.16704	-1.08519	2.35930
C	0.10150	-2.57534	2.43579	-0.01190	-2.54523	2.43087
B	-1.43299	-0.34462	2.34964	-1.46436	-0.27537	2.31912
C	0.43919	1.16771	2.06680	0.44706	1.16146	2.06255
C	-1.88190	2.31076	2.04499	-1.81743	2.38097	2.00313
C	-1.00896	1.11996	2.09665	-0.99203	1.16339	2.07026
C	-3.16676	-2.09991	1.48283	-3.25538	-1.96763	1.47631
H	-2.33838	-2.66684	1.04621	-2.45328	-2.56091	1.04395
C	2.22147	2.62558	3.07293	2.25213	2.55869	3.07616
H	2.41072	1.84283	3.80977	2.39838	1.78209	3.81858
C	1.00874	3.45137	1.16606	1.11346	3.39862	1.15077
H	0.24956	3.32345	0.39349	0.36787	3.29049	0.37205
C	1.73700	4.63862	1.20877	1.87975	4.55103	1.19216
H	1.53695	5.41189	0.46241	1.72306	5.31730	0.43909
C	2.72108	4.82395	2.18257	2.84451	4.71134	2.17608
H	3.30070	5.75022	2.21491	3.45268	5.61023	2.20835
C	2.95490	3.81107	3.11641	3.02311	3.70919	3.11954
H	3.71793	3.94277	3.88853	3.77161	3.82155	3.89838
C	-1.65924	3.43694	2.86510	-1.56065	3.49580	2.81049
H	-0.80904	3.43229	3.54972	-0.72080	3.46584	3.49555
C	-3.84396	3.46592	1.13736	-3.71701	3.58991	1.08036
H	-4.69335	3.45910	0.44856	-4.55686	3.60815	0.39183
C	-4.01515	-0.27945	2.79352	-4.02885	-0.11621	2.75936
H	-3.85717	0.62901	3.38041	-3.83692	0.78788	3.32896
C	-3.00521	2.35395	1.19435	-2.92391	2.45651	1.15041
H	-3.22827	1.49141	0.56189	-3.17131	1.60298	0.52750
C	-0.73396	-3.37976	3.24082	-0.87314	-3.30999	3.22840
H	-1.49413	-2.88888	3.85208	-1.62143	-2.79455	3.82067
C	-5.31429	-0.75299	2.60977	-5.33637	-0.54322	2.58831
H	-6.15449	-0.21862	3.06330	-6.14976	0.02243	3.03475
C	-3.59187	4.57839	1.94373	-3.43279	4.69086	1.87518
H	-4.24270	5.45545	1.89984	-4.04774	5.58399	1.82142
C	-4.46432	-2.57234	1.27650	-4.56101	-2.39459	1.28314
H	-4.62073	-3.46719	0.66721	-4.75094	-3.28521	0.69074
C	-2.49693	4.54996	2.81265	-2.35201	4.63054	2.74495
H	-2.28929	5.40812	3.45819	-2.11904	5.47977	3.38104
C	-5.54985	-1.89803	1.84090	-5.61253	-1.68153	1.84085
H	-6.56941	-2.26195	1.68691	-6.63772	-2.01021	1.69740

C	-0.61291	-4.76761	3.25370	-0.79222	-4.69166	3.25731
H	-1.28149	-5.35899	3.88599	-1.48000	-5.25325	3.88335
C	1.08102	-3.24270	1.66726	0.94800	-3.24139	1.68415
H	1.76257	-2.66428	1.03968	1.64560	-2.69217	1.06083
C	0.35079	-5.40707	2.46570	0.15561	-5.36162	2.49274
H	0.43984	-6.49641	2.47342	0.21376	-6.44553	2.51298
C	1.20073	-4.63159	1.67345	1.02858	-4.62404	1.70618
H	1.95935	-5.10060	1.04070	1.77512	-5.11775	1.09088
C	2.42309	-0.44830	2.38677	2.36306	-0.50374	2.39770
C	3.40010	0.09808	1.53542	3.35325	0.00286	1.55497
H	3.09291	0.73763	0.70709	3.07226	0.64223	0.72668
C	2.86215	-1.27451	3.43955	2.76385	-1.33014	3.45083
H	2.12239	-1.70486	4.11756	2.01039	-1.73131	4.11989
C	4.75493	-0.17175	1.71646	4.68935	-0.30560	1.74555
H	5.47816	0.26354	1.02178	5.42582	0.09967	1.05831
C	4.21754	-1.54861	3.62080	4.09996	-1.64325	3.64249
H	4.52905	-2.19802	4.44376	4.38370	-2.29270	4.46558
C	5.17329	-1.00241	2.75957	5.07126	-1.13551	2.79036
H	6.23451	-1.22413	2.89961	6.11706	-1.38701	2.93862
C	-1.15223	-2.43761	-2.29825	-1.19887	-2.40958	-2.29139
C	-0.89096	0.11602	-2.47329	-0.85124	0.11510	-2.47402
C	2.93424	0.98180	-2.02451	2.97716	0.86126	-2.00824
C	0.14519	1.10972	-2.28461	0.20696	1.07235	-2.29435
C	-0.06767	2.57082	-2.39519	0.04043	2.53342	-2.41671
B	1.48231	0.36571	-2.07213	1.50930	0.29357	-2.06918
C	-0.33618	-1.20862	-2.34504	-0.34486	-1.21147	-2.32814
C	1.98285	-2.29355	-2.03074	1.91842	-2.36839	-1.98435
C	1.08247	-1.12780	-2.05804	1.06256	-1.17619	-2.03568
C	3.23006	2.17691	-1.33660	3.29649	2.04755	-1.33750
H	2.43213	2.69738	-0.80362	2.51189	2.59669	-0.82899
C	-2.20841	-2.67150	-3.20383	-2.22688	-2.61060	-3.21945
H	-2.39299	-1.94512	-3.99765	-2.36301	-1.88656	-4.01524
C	-0.92739	-3.40830	-1.29825	-1.03623	-3.36963	-1.28602
H	-0.08875	-3.29686	-0.60579	-0.22153	-3.27933	-0.57374
C	-1.74018	-4.53580	-1.17901	-1.88383	-4.46191	-1.18469
H	-1.54068	-5.25012	-0.37605	-1.73333	-5.17252	-0.37781
C	-2.79368	-4.73622	-2.07183	-2.90868	-4.63312	-2.10176
H	-3.43439	-5.61701	-1.98298	-3.57589	-5.48603	-2.02703
C	-3.01400	-3.80128	-3.09050	-3.06761	-3.70494	-3.12477
H	-3.82771	-3.95463	-3.80460	-3.86012	-3.83455	-3.85585
C	1.83299	-3.37891	-2.92072	1.73822	-3.45256	-2.85246
H	1.01802	-3.35914	-3.64739	0.93437	-3.41689	-3.57980
C	3.93951	-3.43526	-1.10149	3.81449	-3.55228	-1.02929
H	4.75949	-3.43920	-0.37802	4.62344	-3.57276	-0.30484
C	4.00999	0.36354	-2.69643	4.03354	0.20217	-2.64898

H	3.82884	-0.56203	-3.24919	3.83166	-0.72006	-3.18530
C	3.06889	-2.34885	-1.13249	2.98665	-2.44491	-1.08339
H	3.22531	-1.51631	-0.44385	3.16425	-1.61267	-0.41141
C	0.78636	3.33728	-3.21305	0.91578	3.26050	-3.22998
H	1.57730	2.82426	-3.76361	1.68797	2.72192	-3.76817
C	5.30168	0.89312	-2.66655	5.33255	0.68756	-2.60746
H	6.11005	0.38038	-3.19675	6.12663	0.14498	-3.11361
C	3.76020	-4.51038	-1.97741	3.60668	-4.62634	-1.88474
H	4.43822	-5.36732	-1.95219	4.25095	-5.49914	-1.84195
C	4.51864	2.70923	-1.29032	4.59226	2.53598	-1.27910
H	4.70117	3.62563	-0.72141	4.79481	3.44838	-0.72508
C	2.70355	-4.46790	-2.89121	2.56544	-4.56295	-2.80037
H	2.55393	-5.29367	-3.59295	2.39360	-5.38835	-3.48568
C	5.56657	2.06685	-1.95554	5.62153	1.85542	-1.91506
H	6.57994	2.47694	-1.92103	6.63978	2.23151	-1.87164
C	0.65073	4.72204	-3.30913	0.82446	4.63897	-3.33683
H	1.33762	5.28603	-3.94653	1.52664	5.17283	-3.97083
C	-1.07140	3.26537	-1.69115	-0.93655	3.25797	-1.72630
H	-1.75445	2.71941	-1.03680	-1.63494	2.74217	-1.07554
C	-0.34626	5.38976	-2.59202	-0.14816	5.33794	-2.63445
H	-0.44551	6.47620	-2.66080	-0.21343	6.41895	-2.71186
C	-1.20959	4.64970	-1.78086	-1.03076	4.63627	-1.82617
H	-1.98680	5.14505	-1.19254	-1.78972	5.15674	-1.24960
C	-2.33907	0.42002	-2.51202	-2.28526	0.45907	-2.51167
C	-3.23516	-0.17796	-1.60066	-3.18662	-0.10650	-1.60180
H	-2.90738	-0.97799	-0.92645	-2.88155	-0.90935	-0.93244
C	-2.86390	1.38545	-3.39451	-2.78075	1.42808	-3.39052
H	-2.19206	1.86590	-4.10772	-2.10174	1.88495	-4.10124
C	-4.58183	0.18588	-1.54518	-4.51434	0.29150	-1.54347
H	-5.22928	-0.28921	-0.80404	-5.16742	-0.15965	-0.80302
C	-4.20986	1.73928	-3.35256	-4.10752	1.81597	-3.34655
H	-4.58820	2.49246	-4.04888	-4.46468	2.57198	-4.03935
C	-5.07530	1.15127	-2.42240	-4.98032	1.25909	-2.41763
H	-6.12762	1.44341	-2.38584	-6.01692	1.57845	-2.37953

Table S9. Normal mode frequencies (cm^{-1}) and transition intensities (km mol^{-1}) of the vibrational modes of **1-PBE**.

Mode	Frequency (cm^{-1})	Transition intensity (km mol^{-1})	Mode	Frequency (cm^{-1})	Transition intensity (km mol^{-1})	Mode	Frequency (cm^{-1})	Transition intensity (km mol^{-1})
1	13.618	0.751	120	725.549	10.232	239	1264.483	31.344
2	24.144	0.181	121	725.927	12.957	240	1278.342	2.231
3	25.629	0.166	122	730.005	16.494	241	1279.631	1.262
4	29.330	0.031	123	731.142	9.359	242	1280.357	1.520
5	29.541	0.164	124	746.077	2.942	243	1281.343	1.705
6	31.323	0.350	125	747.638	0.976	244	1282.650	2.246
7	32.207	0.197	126	751.443	5.788	245	1284.385	0.808
8	37.325	1.075	127	755.446	8.256	246	1286.306	5.757
9	40.000	0.801	128	759.224	12.752	247	1289.557	5.617
10	40.576	1.176	129	760.197	24.317	248	1293.258	2.459
11	42.904	1.357	130	761.569	14.778	249	1296.491	9.180
12	45.033	0.115	131	762.928	18.700	250	1307.327	2.021
13	49.650	0.021	132	778.276	17.171	251	1311.470	7.991
14	51.249	0.058	133	780.011	23.301	252	1324.172	1.543
15	52.928	0.549	134	826.261	0.700	253	1325.323	0.649
16	55.547	0.224	135	828.014	0.536	254	1328.122	0.227
17	56.164	0.350	136	829.832	0.850	255	1328.571	4.925
18	57.753	0.010	137	830.933	0.664	256	1329.157	5.228
19	60.000	0.217	138	831.904	0.789	257	1330.933	1.561
20	62.770	2.966	139	832.539	0.646	258	1331.833	0.207
21	75.173	0.335	140	833.176	0.544	259	1332.290	2.988
22	77.843	3.125	141	833.873	1.065	260	1334.900	0.545
23	79.870	0.176	142	836.468	0.369	261	1335.332	1.646
24	82.410	0.102	143	838.692	1.302	262	1343.561	9.792
25	84.512	0.101	144	880.094	2.824	263	1346.709	14.385
26	93.777	0.047	145	880.986	2.227	264	1351.649	10.198
27	96.056	0.238	146	886.322	4.584	265	1353.686	189.023
28	101.202	0.906	147	886.544	1.928	266	1370.348	30.682
29	105.743	3.118	148	886.989	0.363	267	1376.485	13.324
30	108.747	1.185	149	888.253	3.402	268	1411.427	3.812
31	114.694	1.047	150	888.613	5.644	269	1412.766	6.879
32	140.566	4.457	151	889.303	1.550	270	1423.416	0.599
33	140.978	0.297	152	891.434	0.814	271	1424.396	6.609
34	152.034	0.746	153	892.465	13.221	272	1424.562	2.366
35	162.174	2.063	154	893.099	14.736	273	1425.297	2.253
36	167.027	0.241	155	893.608	0.344	274	1426.720	7.706
37	172.612	3.808	156	909.188	0.260	275	1427.543	11.986
38	174.561	4.714	157	912.978	0.433	276	1428.029	10.611
39	198.271	11.290	158	926.218	0.751	277	1429.609	4.450
40	201.311	14.209	159	930.282	1.098	278	1465.407	9.074
41	202.163	1.890	160	932.572	0.941	279	1469.201	9.750
42	217.865	0.016	161	933.522	0.986	280	1469.711	11.964
43	218.835	0.113	162	935.692	1.043	281	1470.937	22.344
44	220.457	1.111	163	935.862	0.846	282	1471.710	77.459
45	221.847	0.538	164	937.332	0.862	283	1472.426	57.531

46	224.822	0.307	165	937.606	1.121	284	1473.737	91.297
47	227.713	0.631	166	940.099	0.453	285	1475.761	42.424
48	234.161	0.679	167	944.529	0.322	286	1477.648	38.960
49	235.436	0.213	168	945.164	1.071	287	1481.044	23.630
50	241.850	1.348	169	948.504	1.569	288	1552.529	0.664
51	243.669	0.134	170	950.709	0.757	289	1556.655	2.448
52	244.445	0.269	171	951.726	5.048	290	1557.115	0.508
53	247.050	0.063	172	953.566	0.431	291	1558.310	1.505
54	261.343	1.191	173	953.935	0.583	292	1558.754	3.839
55	263.608	1.202	174	954.490	3.434	293	1559.753	0.888
56	276.227	0.483	175	955.371	0.871	294	1560.578	1.744
57	278.477	0.264	176	957.630	0.736	295	1562.040	1.772
58	355.076	9.971	177	959.171	1.583	296	1564.199	1.766
59	365.273	22.381	178	984.101	0.124	297	1566.834	1.186
60	395.986	0.117	179	984.667	0.180	298	1579.083	13.012
61	399.250	0.104	180	985.268	0.578	299	1584.070	13.754
62	402.446	0.140	181	986.044	0.068	300	1585.138	8.283
63	403.533	2.313	182	986.535	2.352	301	1586.933	38.059
64	404.786	4.763	183	986.669	0.087	302	1586.981	68.397
65	405.531	1.566	184	988.261	0.048	303	1588.002	17.067
66	407.484	2.021	185	988.952	0.621	304	1588.387	103.618
67	408.360	0.452	186	989.217	0.017	305	1590.115	27.221
68	409.605	1.116	187	990.519	0.325	306	1591.301	12.777
69	410.949	1.600	188	1002.558	7.415	307	1593.460	21.853
70	411.265	1.949	189	1003.048	1.140	308	3034.419	7.807
71	412.464	1.710	190	1021.924	27.785	309	3057.728	2.073
72	416.541	8.649	191	1022.144	34.691	310	3057.906	5.214
73	422.326	4.398	192	1022.758	4.428	311	3063.702	0.990
74	477.813	0.028	193	1022.963	3.025	312	3064.275	4.892
75	478.912	0.035	194	1024.325	0.067	313	3065.232	2.341
76	494.081	0.477	195	1025.099	2.007	314	3066.054	4.902
77	497.087	0.212	196	1025.949	2.006	315	3066.783	2.897
78	502.614	1.097	197	1026.165	0.092	316	3067.286	3.224
79	508.426	2.481	198	1036.469	0.145	317	3068.146	1.566
80	521.968	9.151	199	1037.388	0.036	318	3070.075	14.139
81	524.080	1.117	200	1060.670	0.752	319	3070.409	3.324
82	529.163	4.472	201	1060.852	0.723	320	3074.130	4.442
83	531.745	6.109	202	1065.944	2.657	321	3074.556	11.718
84	539.841	21.055	203	1067.318	0.749	322	3075.668	15.785
85	542.496	6.294	204	1069.848	7.450	323	3076.005	14.223
86	545.458	15.032	205	1070.327	3.535	324	3076.468	6.894
87	550.024	5.174	206	1072.342	10.590	325	3077.519	12.601
88	606.455	1.922	207	1073.715	25.046	326	3077.949	8.426
89	606.815	0.233	208	1074.478	0.729	327	3081.037	23.003
90	607.233	0.957	209	1076.276	5.754	328	3081.231	10.344
91	608.079	0.552	210	1098.488	7.853	329	3083.522	33.033
92	609.111	2.723	211	1100.869	4.407	330	3086.936	15.071
93	610.420	0.087	212	1104.909	4.991	331	3087.342	43.515
94	611.519	0.006	213	1105.669	12.024	332	3087.882	17.512
95	612.047	0.192	214	1138.208	0.811	333	3088.594	12.090

96	612.853	1.847	215	1138.535	0.788	334	3089.483	33.262
97	613.216	1.112	216	1138.783	0.542	335	3090.000	35.757
98	622.085	0.452	217	1138.904	0.853	336	3091.482	40.811
99	623.845	0.037	218	1139.685	0.346	337	3091.597	23.043
100	635.246	3.258	219	1139.775	0.762	338	3094.339	23.564
101	637.118	2.027	220	1140.278	1.516	339	3094.667	41.969
102	637.772	0.482	221	1140.945	0.590	340	3095.132	26.182
103	652.345	0.843	222	1142.450	1.306	341	3096.411	24.776
104	654.602	2.325	223	1142.891	1.577	342	3097.406	11.217
105	657.581	7.041	224	1143.496	1.176	343	3098.354	13.090
106	660.088	11.161	225	1146.564	0.781	344	3099.540	20.124
107	661.177	29.563	226	1165.358	1.776	345	3099.660	12.657
108	662.271	12.116	227	1166.556	2.484	346	3102.065	19.953
109	685.747	0.389	228	1167.231	3.882	347	3103.213	8.115
110	692.063	28.988	229	1167.637	3.447	348	3103.624	21.328
111	692.455	24.343	230	1169.135	6.578	349	3103.982	2.233
112	692.609	4.437	231	1170.214	3.828	350	3104.295	12.589
113	693.379	48.600	232	1171.298	2.289	351	3105.557	13.737
114	694.914	30.030	233	1171.470	4.548	352	3106.204	19.140
115	696.087	18.681	234	1173.084	1.920	353	3106.258	13.772
116	696.193	36.071	235	1173.268	1.823	354	3106.449	9.044
117	697.145	93.803	236	1194.666	1.160	355	3111.286	10.084
118	698.820	27.503	237	1196.521	0.063	356	3112.441	2.285
119	700.787	74.588	238	1253.799	11.425	357	3112.753	3.589

Table S10. Normal mode frequencies (cm^{-1}) and transition intensities (km mol^{-1}) of the vibrational modes of **1-PBE0**.

**Mode**	**Frequency (cm^{-1})**	**Transition intensity (km mol^{-1})**	**Mode**	**Frequency (cm^{-1})**	**Transition intensity (km mol^{-1})**	**Mode**	**Frequency (cm^{-1})**	**Transition intensity (km mol^{-1})**
1	14.799	0.632	120	752.763	8.292	239	1309.753	31.275
2	24.451	0.195	121	753.874	13.140	240	1317.025	1.921
3	26.727	0.131	122	758.700	14.313	241	1319.293	0.358
4	29.744	0.017	123	760.119	6.973	242	1320.924	1.032
5	30.791	0.155	124	779.341	1.610	243	1321.696	0.677
6	32.698	0.642	125	781.117	1.975	244	1322.074	0.954
7	33.277	0.271	126	786.624	5.302	245	1325.410	5.876
8	37.204	0.974	127	789.315	25.620	246	1325.798	1.436
9	40.171	0.456	128	791.673	27.695	247	1328.862	3.796
10	41.996	2.292	129	792.966	8.405	248	1338.481	6.165
11	43.350	0.732	130	793.615	16.247	249	1343.749	24.558
12	45.666	0.167	131	794.920	25.940	250	1356.410	1.136
13	50.605	0.017	132	812.439	24.546	251	1356.973	0.487
14	52.912	0.035	133	814.033	29.536	252	1357.783	1.322
15	54.103	0.825	134	866.640	0.721	253	1358.860	0.486
16	56.823	0.202	135	868.118	0.960	254	1359.394	0.809
17	57.524	0.434	136	868.424	0.410	255	1359.715	1.146
18	59.524	0.003	137	871.133	0.391	256	1360.023	0.722
19	61.509	0.047	138	871.642	1.017	257	1361.358	0.967
20	64.116	3.730	139	872.037	0.431	258	1361.777	2.206
21	76.200	1.237	140	872.794	0.917	259	1363.148	1.169
22	77.962	3.645	141	874.367	0.795	260	1364.181	0.393
23	81.525	0.192	142	876.250	0.572	261	1365.626	4.794
24	84.327	0.189	143	878.971	1.197	262	1395.722	11.256
25	86.053	0.114	144	922.739	1.629	263	1401.778	10.878
26	96.419	0.028	145	924.603	0.426	264	1407.616	16.666
27	99.035	0.197	146	928.881	2.296	265	1409.778	186.686
28	103.980	0.917	147	929.511	3.124	266	1430.250	18.531
29	108.293	3.489	148	934.036	2.478	267	1437.265	6.183
30	112.483	1.225	149	934.405	0.908	268	1462.297	5.458
31	118.425	1.250	150	934.678	6.078	269	1463.530	9.229
32	145.154	2.641	151	937.255	7.118	270	1474.995	0.442
33	146.100	1.600	152	937.307	1.101	271	1476.010	8.546
34	159.155	0.609	153	938.158	2.157	272	1476.444	3.130
35	170.267	2.290	154	940.719	14.099	273	1477.127	1.259
36	175.444	0.247	155	941.404	15.678	274	1479.012	12.399
37	180.617	4.644	156	943.098	1.446	275	1480.024	16.682
38	182.922	3.354	157	946.564	0.499	276	1481.983	12.657
39	205.176	3.116	158	979.418	0.711	277	1483.556	4.295
40	208.353	5.054	159	984.400	1.112	278	1521.316	7.386
41	210.017	21.832	160	985.893	0.446	279	1524.610	2.784
42	225.392	0.054	161	986.210	1.703	280	1527.100	20.431
43	226.105	0.065	162	988.182	0.550	281	1528.588	20.349
44	229.044	1.180	163	988.312	0.938	282	1529.418	83.900
45	229.454	0.105	164	988.952	1.276	283	1529.953	32.323

46	233.492	0.621	165	991.085	0.581	284	1531.336	128.956
47	237.402	0.680	166	992.800	0.487	285	1533.477	80.374
48	242.324	0.787	167	996.729	1.502	286	1537.334	53.974
49	243.835	0.375	168	997.051	0.032	287	1540.997	27.730
50	251.162	1.494	169	1000.003	0.893	288	1622.215	0.664
51	253.442	0.264	170	1000.677	1.393	289	1626.019	0.321
52	254.838	0.177	171	1002.595	0.409	290	1627.168	2.987
53	257.766	0.031	172	1003.747	3.108	291	1628.678	1.913
54	270.616	1.473	173	1004.810	1.236	292	1629.703	0.459
55	272.595	1.067	174	1006.662	1.329	293	1630.290	6.006
56	285.644	0.542	175	1007.150	2.466	294	1630.677	0.993
57	288.275	0.336	176	1008.493	0.661	295	1631.341	1.824
58	371.503	10.786	177	1011.136	1.397	296	1634.917	1.644
59	382.066	27.552	178	1017.541	0.209	297	1637.165	1.166
60	413.451	0.096	179	1018.252	0.289	298	1650.519	10.543
61	416.751	0.180	180	1018.593	0.189	299	1654.897	8.858
62	419.308	0.801	181	1019.115	0.305	300	1655.894	14.399
63	420.266	4.423	182	1019.413	1.066	301	1658.012	72.058
64	421.155	4.309	183	1019.537	0.307	302	1658.345	51.699
65	422.028	1.129	184	1020.710	0.062	303	1659.000	87.514
66	424.833	2.313	185	1021.377	0.125	304	1659.267	30.427
67	425.699	0.153	186	1021.870	0.383	305	1661.605	30.068
68	426.736	0.833	187	1023.039	0.311	306	1662.762	14.183
69	427.618	1.898	188	1037.876	8.854	307	1665.181	21.211
70	428.694	0.217	189	1038.381	0.274	308	3127.429	9.023
71	430.164	1.073	190	1056.971	31.085	309	3146.623	1.369
72	434.203	9.088	191	1057.063	29.994	310	3146.863	4.523
73	440.506	4.707	192	1057.606	4.933	311	3152.736	3.405
74	497.130	0.013	193	1057.841	0.968	312	3152.908	0.858
75	498.423	0.087	194	1059.377	0.059	313	3153.638	1.864
76	514.691	0.450	195	1059.953	1.510	314	3154.355	4.172
77	517.902	0.200	196	1060.669	0.586	315	3155.069	2.575
78	523.230	0.934	197	1060.820	0.381	316	3155.822	2.419
79	529.861	3.014	198	1072.697	0.113	317	3156.591	1.106
80	541.575	9.237	199	1073.076	0.025	318	3158.973	2.509
81	543.148	0.888	200	1096.603	0.573	319	3159.541	12.806
82	547.923	4.214	201	1097.317	0.792	320	3162.654	3.765
83	550.542	5.423	202	1102.476	3.510	321	3163.834	10.959
84	561.664	25.259	203	1103.906	1.289	322	3164.676	14.580
85	564.832	6.792	204	1105.765	11.157	323	3165.315	16.414
86	567.653	13.154	205	1106.082	1.394	324	3166.076	5.706
87	573.763	4.864	206	1108.319	7.286	325	3166.603	7.144
88	625.838	1.923	207	1109.074	30.772	326	3166.786	11.985
89	625.972	0.076	208	1110.312	0.414	327	3170.011	24.485
90	626.435	0.852	209	1112.507	6.241	328	3170.137	5.118
91	627.475	0.400	210	1139.161	8.021	329	3173.055	31.106
92	628.343	2.476	211	1140.744	2.695	330	3176.260	15.200
93	629.681	0.048	212	1145.138	4.381	331	3176.830	33.146
94	630.820	0.007	213	1145.992	10.349	332	3177.272	10.851
95	631.311	0.149	214	1172.232	0.981	333	3177.613	50.332

96	632.065	1.738	215	1172.617	0.316	334	3178.266	9.319
97	632.395	1.063	216	1172.746	2.111	335	3179.999	47.683
98	642.346	0.403	217	1172.919	0.624	336	3180.843	13.041
99	643.607	0.078	218	1173.445	0.642	337	3181.349	38.895
100	657.187	0.089	219	1173.708	0.421	338	3183.381	16.532
101	658.221	0.782	220	1174.077	1.355	339	3183.526	38.325
102	661.975	4.090	221	1174.684	0.199	340	3184.684	23.599
103	674.226	0.564	222	1175.980	1.640	341	3186.434	18.748
104	675.632	1.771	223	1177.574	1.342	342	3187.514	8.500
105	684.401	7.181	224	1186.000	0.157	343	3187.862	7.695
106	685.739	6.412	225	1189.043	0.545	344	3188.697	11.221
107	686.368	35.036	226	1202.684	1.491	345	3189.776	17.700
108	691.231	22.945	227	1204.600	1.655	346	3190.917	16.870
109	718.641	0.560	228	1204.913	3.541	347	3192.983	12.272
110	721.057	15.238	229	1205.206	2.579	348	3193.033	14.347
111	722.494	37.005	230	1206.261	2.377	349	3194.627	0.902
112	722.640	24.623	231	1206.409	3.993	350	3194.943	18.073
113	723.433	35.087	232	1207.041	4.501	351	3195.044	16.729
114	724.886	29.536	233	1208.844	3.183	352	3195.087	2.699
115	725.698	14.398	234	1209.230	1.278	353	3195.349	7.917
116	726.292	21.199	235	1210.366	2.438	354	3195.614	6.377
117	727.170	108.415	236	1241.849	2.071	355	3200.355	8.611
118	729.204	35.840	237	1244.154	0.543	356	3201.792	1.415
119	730.527	102.514	238	1302.789	20.529	357	3203.881	2.959

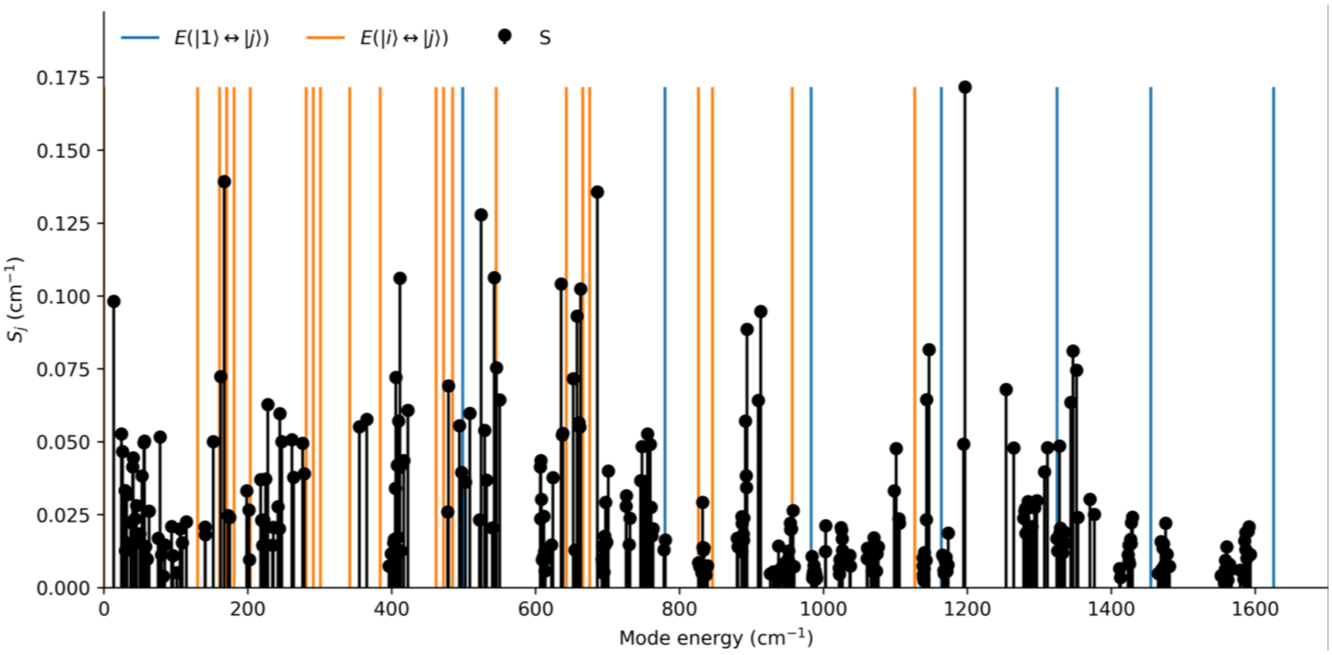

Figure S25. Vibrational coupling strength (S_j)[20] of each vibrational mode of **1-PBE** in the range of 0 to 1700 cm^{-1}. Electronic energy states are shown in blue and transitions between states are given in orange.

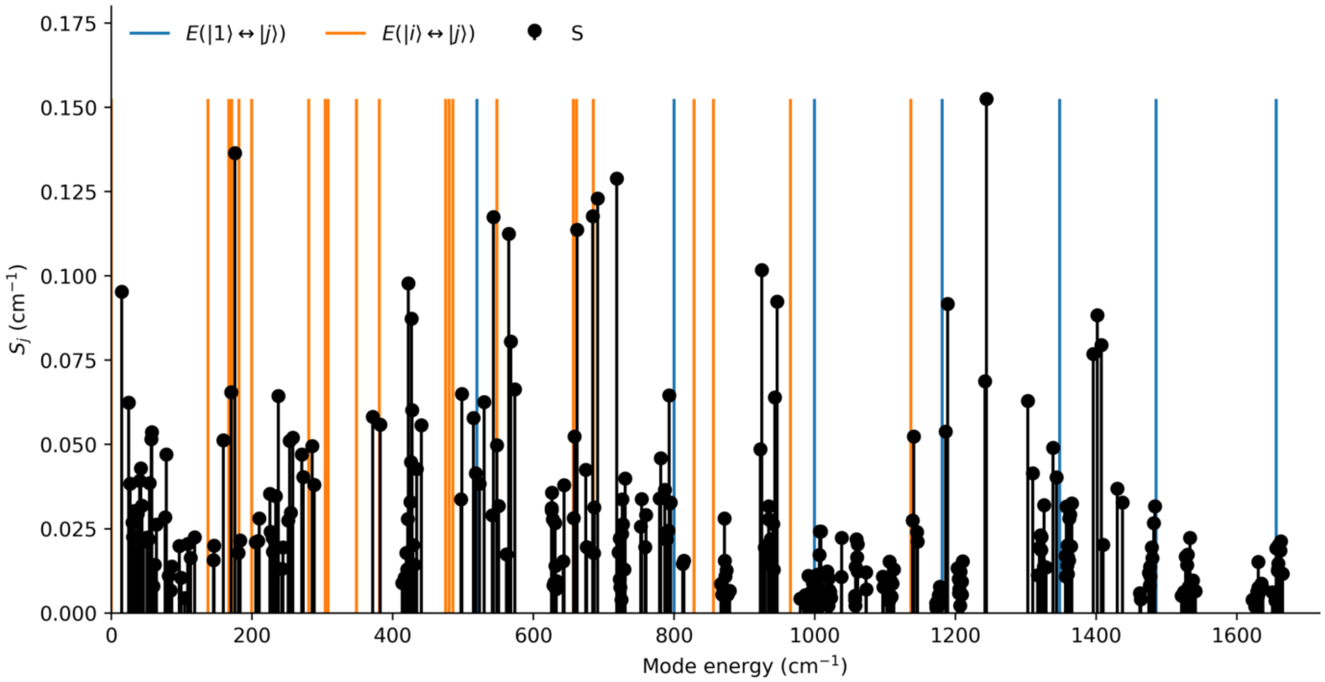

Figure S26. Vibrational coupling strength (S_j)[20] of each vibrational mode of **1-PBE0** in the range of 0 to 1700 cm^{-1}. Electronic energy states are shown in blue and transitions between states are given in orange.

Table S11. Calculated electronic structure of one of the $[Dy(BC_4Ph_5)_2]^-$ anions in **1**·2THF.

**Energy (cm^{-1})**	**CF Energy (cm^{-1})**	g_x	g_y	g_z	θ **(°)**	**Wavefunction**	**$\langle J_z \rangle$**
0.00	0.00	0.00	0.00	20.00	--	100% |±15/2⟩	± 7.50
555.22	567.86	0.00	0.00	17.02	1.95	100% |±13/2⟩	± 6.50
891.77	885.11	0.00	0.00	14.37	1.61	99% |±11/2⟩	± 5.50
1111.88	1110.64	0.03	0.03	11.77	5.41	99% |±9/2⟩	± 4.49
1300.73	1308.39	0.38	0.41	9.05	9.52	97% |±7/2⟩ + 2% |±3/2⟩	± 3.45
1476.28	1485.66	3.35	3.61	5.98	17.28	88% |±5/2⟩ + 7% |±1/2⟩ + 2% |∓3/2⟩	± 2.26
1624.30	1628.71	2.39	5.34	10.84	87.55	73% |±3/2⟩ + 17% |∓1/2⟩ + 6% |∓5/2⟩	± 0.95
1790.59	1787.05	0.36	1.29	18.03	88.96	65% |±1/2⟩ + 16% |∓3/2⟩ + 10% |∓1/2⟩	± 0.18

Table S12. Calculated electronic structure of one of the $[Dy(BC_4Ph_5)_2]^-$ anions in **1**·2THF using higher level methodology.

**Energy (cm^{-1})**	**CF Energy (cm^{-1})**	g_x	g_y	g_z	θ **(°)**	**Wavefunction**	**$\langle J_z \rangle$**
0.00	0.00	0.00	0.00	19.90	--	100% |±15/2⟩	± 7.50
542.59	550.73	0.00	0.00	16.99	2.03	100% |±13/2⟩	± 6.50
860.06	855.64	0.00	0.00	14.35	1.24	99% |±11/2⟩	± 5.50
1070.65	1070.05	0.03	0.03	11.76	5.67	99% |±9/2⟩	± 4.49
1252.31	1257.28	0.41	0.44	9.04	9.52	96% |±7/2⟩ + 2% |±3/2⟩	± 3.44
1419.20	1425.18	3.45	3.73	5.96	17.59	87% |±5/2⟩ + 8% |±1/2⟩ + 2% |∓3/2⟩	± 2.25
1558.69	1561.53	2.37	5.23	10.93	87.59	72% |±3/2⟩ + 17% |∓1/2⟩ + 6% |∓5/2⟩	± 0.94
1715.26	1712.98	0.35	1.22	18.00	88.93	64% |±1/2⟩ + 16% |∓3/2⟩ + 10% |∓1/2⟩	± 0.18

Table S13. Electronic structure of **1-PBE**.

Energy (cm^{-1})	CF Energy (cm^{-1})	g_x	g_y	g_z	θ (°)	Wavefunction	<J_z>
0.00	0.00	0.00	0.00	20.00	--	100% |±15/2⟩	± 7.50
488.59	498.50	0.00	0.00	17.06	1.94	100% |±13/2⟩	± 6.50
784.44	779.74	0.00	0.00	14.42	1.01	99% |±11/2⟩	± 5.50
984.25	982.90	0.02	0.02	11.80	5.04	99% |±9/2⟩	± 4.50
1158.42	1163.86	0.13	0.15	9.05	8.87	97% |±7/2⟩ + 2% |±3/2⟩	± 3.45
1317.09	1324.68	3.66	4.13	5.91	22.98	86% |±5/2⟩ + 9% |±1/2⟩ + 2% |∓3/2⟩	± 2.21
1450.04	1454.95	2.15	5.36	11.36	89.89	70% |±3/2⟩ + 19% |∓1/2⟩ + 8% |∓5/2⟩	± 0.84
1628.98	1625.53	0.24	0.76	18.36	89.65	66% |±1/2⟩ + 20% |∓3/2⟩ + 5% |∓1/2⟩	± 0.10

Table S14. Electronic structure of **1-PBE** using higher level methodology.

Energy (cm^{-1})	CF Energy (cm^{-1})	g_x	g_y	g_z	θ (°)	Wavefunction	<J_z>
0.00	0.00	0.00	0.00	19.89	--	100% |±15/2⟩	± 7.50
475.46	481.82	0.00	0.00	17.03	1.99	100% |±13/2⟩	± 6.50
755.40	752.32	0.00	0.00	14.39	0.75	99% |±11/2⟩	± 5.50
946.94	946.17	0.02	0.02	11.78	5.29	99% |±9/2⟩	± 4.49
1114.46	1117.95	0.18	0.19	9.04	8.91	97% |±7/2⟩ + 2% |±3/2⟩	± 3.44
1265.34	1270.23	3.79	4.22	5.90	24.31	85% |±5/2⟩ + 10% |±1/2⟩ + 2% |∓3/2⟩	± 2.20
1391.21	1394.44	2.11	5.21	11.44	89.88	70% |±3/2⟩ + 18% |∓1/2⟩ + 8% |∓5/2⟩	± 0.83
1559.99	1557.71	0.23	0.71	18.31	89.63	65% |±1/2⟩ + 20% |∓3/2⟩ + 6% |∓1/2⟩	± 0.10

Table S15. Electronic structure of **1-PBE0**.

Energy (cm^{-1})	CF Energy (cm^{-1})	g_x	g_y	g_z	θ (°)	Wavefunction	<J_z>
0.00	0.00	0.00	0.00	20.00	--	100% |±15/2⟩	± 7.50
508.38	519.61	0.00	0.00	17.05	2.07	100% |±13/2⟩	± 6.50
805.69	800.09	0.00	0.00	14.42	1.13	99% |±11/2⟩	± 5.50
1001.08	999.64	0.02	0.03	11.82	5.65	99% |±9/2⟩	± 4.49
1174.73	1181.03	0.10	0.14	9.07	9.40	97% |±7/2⟩ + 2% |±3/2⟩	± 3.45
1339.36	1347.96	3.46	3.91	5.93	19.90	87% |±5/2⟩ + 9% |±1/2⟩ + 2% |∓3/2⟩	± 2.24
1480.04	1485.07	2.25	5.48	11.05	89.73	71% |±3/2⟩ + 18% |∓1/2⟩ + 7% |∓5/2⟩	± 0.88
1659.86	1656.15	0.28	0.94	18.22	89.83	69% |±1/2⟩ + 20% |∓3/2⟩ + 3% |∓1/2⟩	± 0.11

Table S16. Electronic structure of **1-PBE0** using higher level methodology.

Energy (cm^{-1})	CF Energy (cm^{-1})	g_x	g_y	g_z	θ (°)	Wavefunction	<J_z>
0.00	0.00	0.00	0.00	19.90	--	100% |±15/2⟩	± 7.50
494.97	502.14	0.00	0.00	17.02	2.14	100% |±13/2⟩	± 6.50
775.54	771.88	0.00	0.00	14.39	0.83	99% |±11/2⟩	± 5.50
962.96	962.16	0.02	0.02	11.80	5.92	99% |±9/2⟩	± 4.49
1130.25	1134.28	0.14	0.17	9.06	9.46	97% |±7/2⟩ + 2% |±3/2⟩	± 3.44
1286.92	1292.43	3.58	4.01	5.92	20.89	86% |±5/2⟩ + 9% |±1/2⟩ + 2% |∓3/2⟩	± 2.23
1419.87	1423.15	2.22	5.34	11.14	89.75	71% |±3/2⟩ + 18% |∓1/2⟩ + 7% |∓5/2⟩	± 0.87
1589.24	1586.81	0.27	0.88	18.18	89.82	68% |±1/2⟩ + 20% |∓3/2⟩ + 4% |∓1/2⟩	± 0.11

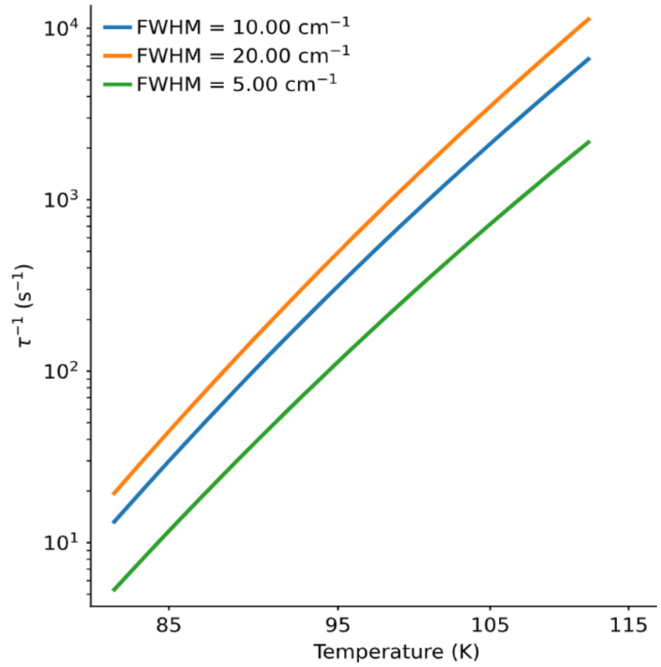

Figure S27. Calculated relaxation rates of **1-PBE** with fixed FWHM linewidths of 5 cm^{-1} (green), 10 cm^{-1} (blue) and 20 cm^{-1} (orange) in the temperature range of 82–112 K.

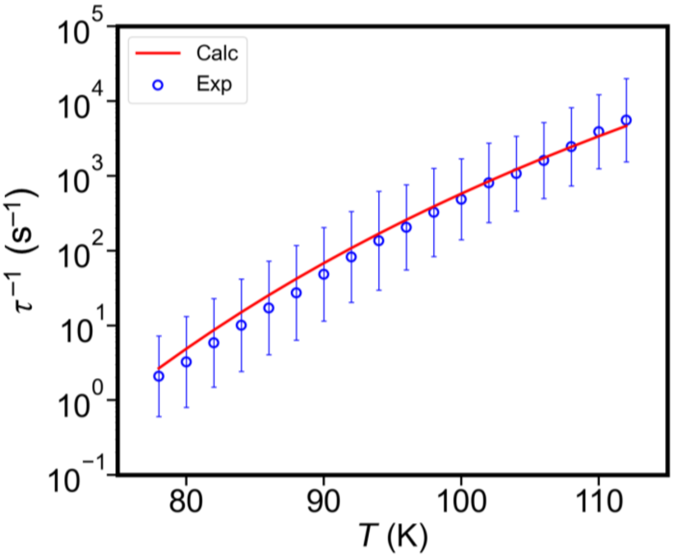

Figure S28. Temperature dependence of calculated relaxation rates for **1-PBE0** (red line, FWHM = 10 cm^{-1}) and experimental relaxation rates for **1** (blue symbols). Error bars are 1σ estimated standard deviations from the generalized Debye model.[12]

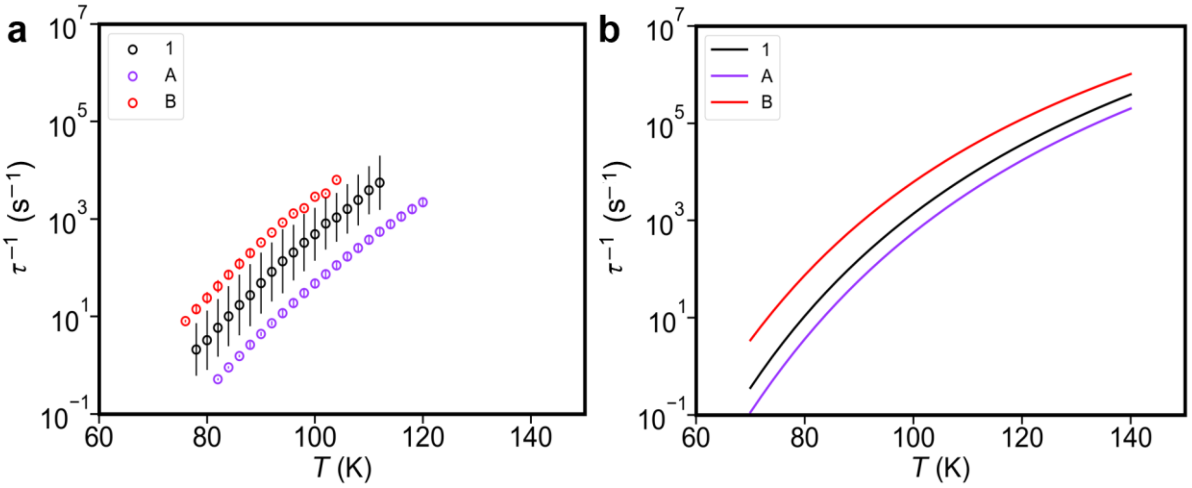

Figure S29. Temperature dependence of (a) experimental and (b) calculated relaxation rates for the **1-PBE** model (black), $[Dy(Cp^{iPr5})(Cp^{Me5})]^+$ (**A**; purple), and $[Dy(Cp^{iPr4})_2]^+$ (**B**; red). Calculations were all performed using a consistent methodology as for **1-PBE** with FWHM = 10 cm^{-1}.

Table S17. Mode-weighted comparison of **1-PBE** with $[Dy(Cp^{iPr5})(Cp^{Me5})]^+$ (**A**) performed at 100 K with FWHM = 10 cm^{-1}.

$\langle \bar{\bar{H}}_{SP} \rangle$	$\langle \bar{\bar{Q}} \rangle$	$\langle \bar{\bar{\rho}} \rangle$	$\langle \bar{\bar{n}} \rangle$	τ^{-1} (s^{-1})	$\tau^{-1}/\tau^{-1}{}_{\mathbf{1}}$
**A**	**1-PBE**	**1-PBE**	**1-PBE**	3.91×10^2	0.45
**1-PBE**	**1-PBE**	**A**	**1-PBE**	8.49×10^2	0.98
**1-PBE**	**1-PBE**	**1-PBE**	**1-PBE**	8.64×10^2	1.00
**1-PBE**	**1-PBE**	**1-PBE**	**A**	8.81×10^2	1.02
**1-PBE**	**A**	**1-PBE**	**1-PBE**	1.22×10^3	1.41
$\langle \bar{\bar{H}}_{SP} \rangle$	$\langle \bar{\bar{Q}} \rangle$	$\langle \bar{\bar{\rho}} \rangle$	$\langle \bar{\bar{n}} \rangle$	τ^{-1} (s^{-1})	$\tau^{-1}/\tau^{-1}{}_{\mathbf{A}}$
**A**	**A**	**1-PBE**	**A**	1.21×10^2	0.22
**A**	**A**	**A**	**1-PBE**	3.40×10^2	0.61
**A**	**A**	**A**	**A**	5.61×10^2	1.00
**A**	**1-PBE**	**A**	**A**	5.91×10^2	1.05
**1-PBE**	**A**	**A**	**A**	2.84×10^3	5.06

Table S18. Mode-weighted comparison of **1-PBE** with [Dy(Cp^{iPr4})$_2$]$^+$ (**B**), performed at 100 K with FWHM = 10 cm^{-1}.

$\langle \bar{\bar{H}}_{SP} \rangle$	$\langle \bar{\bar{Q}} \rangle$	$\langle \bar{\bar{\rho}} \rangle$	$\langle \bar{\bar{n}} \rangle$	τ^{-1} (s^{-1})	$\tau^{-1}/\tau^{-1}{}_{\mathbf{1}}$
**1-PBE**	**1-PBE**	**1-PBE**	**B**	6.64×10^2	0.77
**1-PBE**	**1-PBE**	**1-PBE**	**1-PBE**	8.64×10^2	1.00
**1-PBE**	**1-PBE**	**B**	**1-PBE**	1.02×10^3	1.18
**B**	**1-PBE**	**1-PBE**	**1-PBE**	1.04×10^3	1.20
**1-PBE**	**B**	**1-PBE**	**1-PBE**	8.27×10^3	9.57
$\langle \bar{\bar{H}}_{SP} \rangle$	$\langle \bar{\bar{Q}} \rangle$	$\langle \bar{\bar{\rho}} \rangle$	$\langle \bar{\bar{n}} \rangle$	τ^{-1} (s^{-1})	$\tau^{-1}/\tau^{-1}{}_{\mathbf{B}}$
**B**	**1-PBE**	**B**	**B**	4.16×10^2	0.07
**B**	**B**	**1-PBE**	**B**	3.04×10^3	0.49
**B**	**B**	**B**	**1-PBE**	4.70×10^3	0.76
**B**	**B**	**B**	**B**	6.17×10^3	1.00
**1-PBE**	**B**	**B**	**B**	1.90×10^4	3.08

Table S19. Calculated Löwdin charges for [C_4BPh_5]$^{2-}$, [Cp^{iPr5}]$^-$ and [Cp*]$^-$ ligands.

**[C_4BPh_5]$^{2-}$**	**[Cp^{iPr5}]$^-$**	**[Cp*]$^-$**
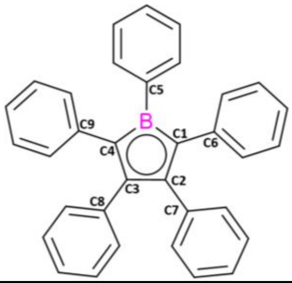	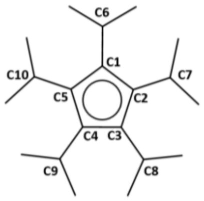	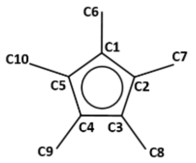

Atom	Löwdin charge	Group	Löwdin charge	Atom	Löwdin charge	Group	Löwdin charge	Atom	Löwdin charge	Group	Löwdin charge
B	−0.37	Ph(C5)	−0.26	C1	−0.12	^{i}Pr(C6)	−0.08	C1	−0.14	Me(C6)	−0.07
C1	−0.09	Ph(C6)	−0.28	C2	−0.12	^{i}Pr(C7)	−0.08	C2	−0.13	Me(C7)	−0.06
C2	−0.08	Ph(C7)	−0.25	C3	−0.12	^{i}Pr(C8)	−0.08	C3	−0.14	Me(C8)	−0.06
C3	−0.08	Ph(C8)	−0.21	C4	−0.12	^{i}Pr(C9)	−0.08	C4	−0.13	Me(C9)	−0.07
C4	−0.09	Ph(C9)	−0.29	C5	−0.12	^{i}Pr(C10)	−0.08	C5	−0.14	Me(C10)	−0.06
C_4B total	−0.71			C_5 total	−0.60			C_5 total	−0.68		

Table S20. Calculated Löwdin charges for [Cp^{ttt}]$^{-}$ and [P(C^{t}BuCMe)$_2$]$^{-}$ ligands.

**[Cp^{ttt}]$^{–}$**				**[P(C^{t}BuMe)$_2$]$^{–}$**			
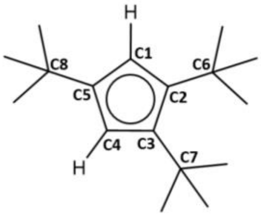				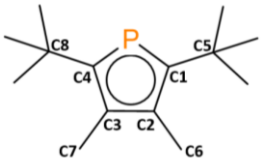			
Atom	Löwdin charge	Group	Löwdin charge	Atom	Löwdin charge	Group	Löwdin charge
C1	−0.29	C6-^{t}Bu	−0.10	P	−0.09	C5-^{t}Bu	−0.07
C2	−0.12	C7-^{t}Bu	−0.10	C1	−0.24	C6-Me	−0.04
C3	−0.12	C8-^{t}Bu	−0.10	C2	−0.10	C7-Me	−0.04
C4	−0.28			C3	−0.10	C8-^{t}Bu	−0.07
C5	−0.11			C4	−0.24		
C_5 total	−0.90			C_4P total	−0.77		

4. References

(1) Herberich, G. E.; Buller, B.; Hessner, B.; Oschmann, W. Derivative Des Borols: II. Pentaphenylborol: Synthese, Reduktion Zum Dianion Und Komplexe von Kobalt Und Platin. *J. Organomet. Chem.* **1980**, *195* (3), 253–259.

(2) Eisch, J. J.; Galle, J. E.; Kozima, S. The Physical and Chemical Consequences of Cyclic Conjugation in Boracyclopolyenes. The Antiaromatic Character of Pentaarylboroles1. *J. Am. Chem. Soc.* **1986**, *108* (3), 379–385.

(3) Niedenzu, K.; Dawson, J. W. Boron-Nitrogen Compounds. III.1,2 Aminoboranes, Part 2: The B-N Bond Character in Substituted Aminoboranes. *J. Am. Chem. Soc.* **1960**, *82* (16), 4223–4228.

(4) Eisch, J. J.; Galle, J. E.; Kozima, S. The Physical and Chemical Consequences of Cyclic Conjugation in Boracyclopolyenes. The Antiaromatic Character of Pentaarylboroles. *J. Am. Chem. Soc.* **1986**, *108* (3), 379–385.

(5) AXS, B. SAINT and APEX 2 Software for CCD Diffractometers. *SAINT and APEX 2 software for CCD diffractometers.* 2006.

(6) Sheldrick, G. M. Sadabs. *University of Göttingen, Germany*. 1996.

(4) Eisch, J. J.; Galle, J. E.; Kozima, S. The Physical and Chemical Consequences of Cyclic Conjugation in Boracyclopolyenes. The Antiaromatic Character of Pentaarylboroles. *J. Am. Chem. Soc.* **1986**, *108* (3), 379–385.

(8) Sheldrick, G. M. Crystal Structure Refinement with SHELXL. *Acta Crystallographica Section C: Structural Chemistry*. January 2015, pp 3–8.

(9) Dolomanov, O. V; Bourhis, L. J.; Gildea, R. J.; Howard, J. A. K.; Puschmann, H. OLEX2: A Complete Structure Solution, Refinement and Analysis Program. *J. Appl. Crystallogr.* **2009**, *42* (2), 339–341.

(10) Hirshfeld, F. L. Can X-Ray Data Distinguish Bonding Effects from Vibrational Smearing? *Acta Crystallography Section A* **1976**, *32* (2), 239–244.

(11) Bain, G. A.; Berry, J. F. Diamagnetic Corrections and Pascal's Constants. *J. Chem. Ed.* **2008**, *85* (4), 1–5.

(12) Hilgar, J. D.; Butts, A. K.; Rinehart, J. D. A Method for Extending AC Susceptometry to Long-Timescale Magnetic Relaxation. *Phys. Chem. Chem. Phys.* **2019**, *21* (40), 22302–22307.

(13) Reta, D.; Chilton, N. F. Uncertainty Estimates for Magnetic Relaxation Times and Magnetic Relaxation Parameters. *Phys. Chem. Chem. Phys.* **2019**, *21* (42), 23567–23575.

(14) Frisch, M. J.; Trucks, G. W.; Schlegel, H. B.; Scuseria, G. E.; Robb, M. A.; Cheeseman, J. R.; Scalmani, G.; Barone, V.; Mennucci, B.; Petersson, G. A.; Nakatsuji, H.; Caricato, M.; Li, X.; Hratchian, H. P.; Izmaylov, A. F.; Bloino, J.; Zheng, G.; Sonnenberg, J. L.; Hada, M.; Ehara, M.; Toyota, K.; Fukuda, R.; Hasegawa, J.; Ishida, M.; Nakajima, T.; Honda, Y.; Kitao, O.; Nakai, H.; Vreven, T.; Montgomery, J. A., Jr.; Peralta, J. E.; Ogliaro, F.; Bearpark, M.; Heyd, J. J.; Brothers, E.; Kudin, K. N.; Staroverov, V. N.; Kobayashi, R.; Normand, J.; Raghavachari, K.; Rendell, A.; Burant, J. C.; Iyengar, S. S.; Tomasi, J.; Cossi, M.; Rega, N.; Millam, J. M.; Klene, M.; Knox, J. E.; Cross, J. B.; Bakken, V.; Adamo, C.; Jaramillo, J.; Gomperts, R.; Stratmann, R. E.; Yazyev, O.; Austin, A. J.; Cammi, R.; Pomelli, C.; Ochterski, J. W.; Martin, R. L.; Morokuma, K.; Zakrzewski, V. G.; Voth, G. A.; Salvador, P.; Dannenberg, J. J.; Dapprich, S.; Daniels, A. D.; Farkas, Ö.; Foresman, J. B.; Ortiz, J. V.; Cioslowski, J.; Fox, D. J. Gaussian, Inc., Wallingford CT, **2009**.

(15) Löwdin, P. On the Non-Orthogonality Problem Connected with the Use of Atomic Wave Functions in the Theory of Molecules and Crystals. *J. Chem. Phys.* **1950**, *18* (3), 365–375.

(16) Rassolov, V. A.; Pople, J. A.; Ratner, M. A.; Windus, T. L. 6-31G* Basis Set for Atoms K through Zn. *J. Chem. Phys.* **1998**, *109* (4), 1223–1229.

(17) Fdez. Galván, I.; Vacher, M.; Alavi, A.; Angeli, C.; Aquilante, F.; Autschbach, J.; Bao, J. J.; Bokarev, S. I.; Bogdanov, N. A.; Carlson, R. K.; Chibotaru, L. F.; Creutzberg, J.; Dattani, N.; Delcey, M. G.; Dong, S. S.; Dreuw, A.; Freitag, L.; Frutos, L. M.; Gagliardi, L.; Gendron, F.; Giussani, A.; González, L.; Grell, G.; Guo, M.; Hoyer, C. E.; Johansson, M.; Keller, S.; Knecht, S.; Kovačević, G.; Källman, E.; Li Manni, G.; Lundberg, M.; Ma, Y.; Mai, S.; Malhado, J. P.; Malmqvist, P. Å.; Marquetand, P.; Mewes, S. A.; Norell, J.; Olivucci, M.; Oppel, M.; Phung, Q. M.; Pierloot, K.; Plasser, F.; Reiher, M.; Sand, A. M.; Schapiro, I.; Sharma, P.; Stein, C. J.; Sørensen, L. K.; Truhlar, D. G.; Ugandi, M.; Ungur, L.; Valentini, A.;

Vancoillie, S.; Veryazov, V.; Weser, O.; Wesołowski, T. A.; Widmark, P.-O.; Wouters, S.; Zech, A.; Zobel, J. P.; Lindh, R. OpenMolcas: From Source Code to Insight. *J. Chem. Theory Comput.* **2019**, *15* (11), 5925–5964.
(18) Roos, B. O.; Lindh, R.; Malmqvist, P.-Å.; Veryazov, V.; Widmark, P.-O. Main Group Atoms and Dimers Studied with a New Relativistic ANO Basis Set. *J. Phys. Chem. A* **2004**, *108* (15), 2851–2858.
(19) Ungur, L.; Chibotaru, L. F. Ab Initio Crystal Field for Lanthanides. *Chem. Eur. J.* **2017**, *23* (15), 3708–3718.
(20) Reta, D.; Kragskow, J. G. C.; Chilton, N. F. Ab Initio Prediction of High-Temperature Magnetic Relaxation Rates in Single-Molecule Magnets. *J. Am. Chem. Soc.* **2021**, *143* (15), 5943–5950.
(21) Kragskow, J. G. C.; Marbey, J.; Buch, C. D.; Nehrkorn, J.; Ozerov, M.; Piligkos, S.; Hill, S.; Chilton, N. F. Analysis of Vibronic Coupling in a 4f Molecular Magnet with FIRMS. *Nat. Commun.* **2022**, *13* (1), 825.
(22) Layfield, R. A.; Tong, M.-L.; Day, B. M.; Chen, Y.-C.; Guo, F.-S.; Mansikkamäki, A. Magnetic Hysteresis up to 80 Kelvin in a Dysprosium Metallocene Single-Molecule Magnet. *Science* **2018**, *362* (6421), 1400–1403.
(23) McClain, K. R.; Gould, C. A.; Chakarawet, K.; Teat, S. J.; Groshens, T. J.; Long, J. R.; Harvey, B. G. High-Temperature Magnetic Blocking and Magneto-Structural Correlations in a Series of Dysprosium(Iii) Metallocenium Single-Molecule Magnets. *Chem. Sci.* **2018**, *9* (45), 8492–8503.
(24) So, C.-W.; Watanabe, D.; Wakamiya, A.; Yamaguchi, S. Synthesis and Structural Characterization of Pentaarylboroles and Their Dianions. *Organometallics* **2008**, *27* (14), 3496–3501.
